# Seascape genomics as a new tool to empower coral reef conservation strategies: An example on north‐western Pacific *Acropora digitifera*

**DOI:** 10.1111/eva.12944

**Published:** 2020-03-19

**Authors:** Oliver Selmoni, Estelle Rochat, Gael Lecellier, Veronique Berteaux‐Lecellier, Stéphane Joost

**Affiliations:** ^1^ Laboratory of Geographic Information Systems (LASIG) School of Architecture, Civil and Environmental Engineering Ecole Polytechnique Fédérale de Lausanne (EPFL) Lausanne Switzerland; ^2^ UMR250/9220 ENTROPIE IRD‐CNRS‐UR Labex CORAIL Noumea New Caledonia; ^3^ UVSQ Université de Paris‐Saclay Versailles France

**Keywords:** *Acropora digitifera*, climate change, conservation genomics, coral bleaching, coral reefs, local adaptation, Ryukyu Archipelago, seascape genomics

## Abstract

Coral reefs are suffering a major decline due to the environmental constraints imposed by climate change. Over the last 20 years, three major coral bleaching events occurred in concomitance with anomalous heatwaves, provoking a severe loss of coral cover worldwide. The conservation strategies for preserving reefs, as they are implemented now, cannot cope with global climatic shifts. Consequently, researchers are advocating for preservation networks to be set‐up to reinforce coral adaptive potential. However, the main obstacle to this implementation is that studies on coral adaption are usually hard to generalize at the scale of a reef system. Here, we study the relationships between genotype frequencies and environmental characteristics of the sea (seascape genomics), in combination with connectivity analysis, to investigate the adaptive potential of a flagship coral species of the Ryukyu Archipelago (Japan). By associating genotype frequencies with descriptors of historical environmental conditions, we discovered six genomic regions hosting polymorphisms that might promote resistance against heat stress. Remarkably, annotations of genes in these regions were consistent with molecular roles associated with heat responses. Furthermore, we combined information on genetic and spatial distances between reefs to predict connectivity at a regional scale. The combination of these results portrayed the adaptive potential of this population: we were able to identify reefs carrying potential heat stress adapted genotypes and to understand how they disperse to neighbouring reefs. This information was summarized by objective, quantifiable and mappable indices covering the whole region, which can be extremely useful for future prioritization of reefs in conservation planning. This framework is transferable to any coral species on any reef system and therefore represents a valuable tool for empowering preservation efforts dedicated to the protection of coral reefs in warming oceans.

## 1 INTRODUCTION

Coral reefs are suffering a severe decline due to the effects of climate change (Hughes et al., 2017). Loss of reef is already showing catastrophic consequences for marine wildlife that depend on these structures (Pratchett, Thompson, Hoey, Cowman, & Wilson, 2018), with disastrous aftermaths expected for human economies as well (Moberg & Folke, 1999). One of the major threats to the persistence of these ecosystems is coral bleaching (Bellwood, Hughes, Folke, & Nyström, 2004): a physiological response induced by environmental stress that provokes hard skeleton corals, the cornerstone of reefs, to separate from the symbiotic microbial algae essential for their survival (Mydlarz, McGinty, & Harvell, 2010).

Over the last 20 years, episodes of coral bleaching struck worldwide and resulted in a local coral cover loss of up to 50% (Hughes et al., 2017, 2018). Heat stress is considered the main driver of coral bleaching (Hughes et al., 2017), but additional stressors causing coral decline were also identified (e.g. ocean acidification, water eutrophication, sedimentation and overfishing; Anthony, Kline, Diaz‐Pulido, Dove, & Hoegh‐Guldberg, 2008; Ateweberhan et al., 2013; Maina, Venus, McClanahan, & Ateweberhan, 2008).

Conservation efforts to mitigate the threat of coral bleaching tend to focus on restoring reefs that have undergone severe losses, as well as limit the impact of future bleaching events (Baums, 2008; Bellwood et al., 2004; Young, Schopmeyer, & Lirman, 2012). To achieve these aims, two main strategies are currently used: establish marine protected areas (MPAs) at reefs and develop coral nurseries (Baums, 2008; Bellwood et al., 2004; Young et al., 2012). MPAs are designated zones in which human access and activities are restricted in order to alleviate the effects of local anthropogenic stressors (Lester et al., 2009). Coral nurseries are underwater gardens of colonies that can then be transplanted to restore damaged reefs (Baums, 2008; Young et al., 2012). For both conservation strategies, researchers advocate the use of methods that account for demographic connectivity such that the location of a conservation measure can also promote resistance and resilience for neighbouring sites (Baums, 2008; Krueck et al., 2017; Lukoschek, Riginos, & Oppen, 2016; Palumbi, 2003; Shanks, Grantham, & Carr, 2003). Despite the observed beneficial effects of these conservation policies worldwide (Cinner et al., 2016; Rodgers et al., 2017; Selig & Bruno, 2010), these solutions do not confer resistance against the heat stress associated with the last mass bleaching events (Baums, 2008; Hughes et al., 2017). Coral reefs that had experienced previous heat stress were found to be more resistant to subsequent heatwaves (Hughes et al., 2019; Krueger et al., 2017; Penin, Vidal‐Dupiol, & Adjeroud, 2013; Thompson & van Woesik, 2009), but to date this information is neglected in conservation actions (Baums, 2008; Maina, McClanahan, Venus, Ateweberhan, & Madin, 2011; OECD, 2017). There is an urgent need to understand whether these observations are due to evolutionary processes and, if so, to determine how the underlying adaptive potential could be included in predictions of climate change responses and in conservation programs (Baums, 2008; Logan, Dunne, Eakin, & Donner, 2014; Maina et al., 2011; Van Oppen, Oliver, Putnam, & Gates, 2015).

To this end, seascape genomics tools are likely to play an important role. Seascape genomics is the marine counterpart of landscape genomics, a branch of population genomics that investigates adaptive potential through field‐based experiments (Balkenhol et al., 2017). Samples that are collected across a landscape are genotyped using next‐generation‐sequencing techniques, describing thousands of genetic variants, while simultaneously the environmental variables of the study area are characterized, usually using remote sensing data to describe specific local climatic conditions (Leempoel et al., 2017). Genomics and environmental information are then combined to detect genetic polymorphisms associated with particular conditions (i.e*.* potentially adaptive genotypes against a specific condition; Rellstab, Gugerli, Eckert, Hancock, & Holderegger, 2015). This approach has been applied to many terrestrial species and is increasingly being used to analyse marine systems in what is referred to as *seascape genomics* (exhaustively reviewed in Riginos, Crandall, Liggins, Bongaerts, & Treml, 2016). To our knowledge, no seascape genomics experiment has yet been applied to reef corals. In fact, adaptation of these species has been mostly studied via transplantation assays coupled with aquarium conditioning, which is a time‐ and resource‐demanding approach that is often restricted to a couple of reefs experiencing contrasting conditions (Howells, Berkelmans, Oppen, Willis, & Bay, 2013; Krueger et al., 2017; Palumbi, Barshis, Traylor‐Knowles, & Bay, 2014; Sampayo et al., 2016; Ziegler, Seneca, Yum, Palumbi, & Voolstra, 2017). Genotype–environment association studies have also been conducted on corals, but have used either a limited number of markers (<10 Single Nucleotide Polymorphism, SNPs) in Lundgren, Vera, Peplow, Manel, & van Oppen, 2013), a restricted number of locations (two in Bay & Palumbi, 2014), or focused on populations with restricted gene flow (Thomas, Kennington, Evans, Kendrick, & Stat, 2017). Contrary to these previous studies, a seascape genomics approach should cover ecologically meaningful spatial scales and be able to distinguish the pressures caused from different climatic conditions, as well as account for confounding effects of demographic processes (Balkenhol et al., 2017). Of note, recent studies showed that combining population genomics analyses with demographic simulations allows to estimate adaptive potential in corals and provide valuable information for reef preservation (Matz, Treml, Aglyamova, & Bay, 2018; Matz, Treml, & Haller, 2019). A similar approach can be used to transpose findings of seascape genomics studies to inform conservation strategies.

**Figure 1:**
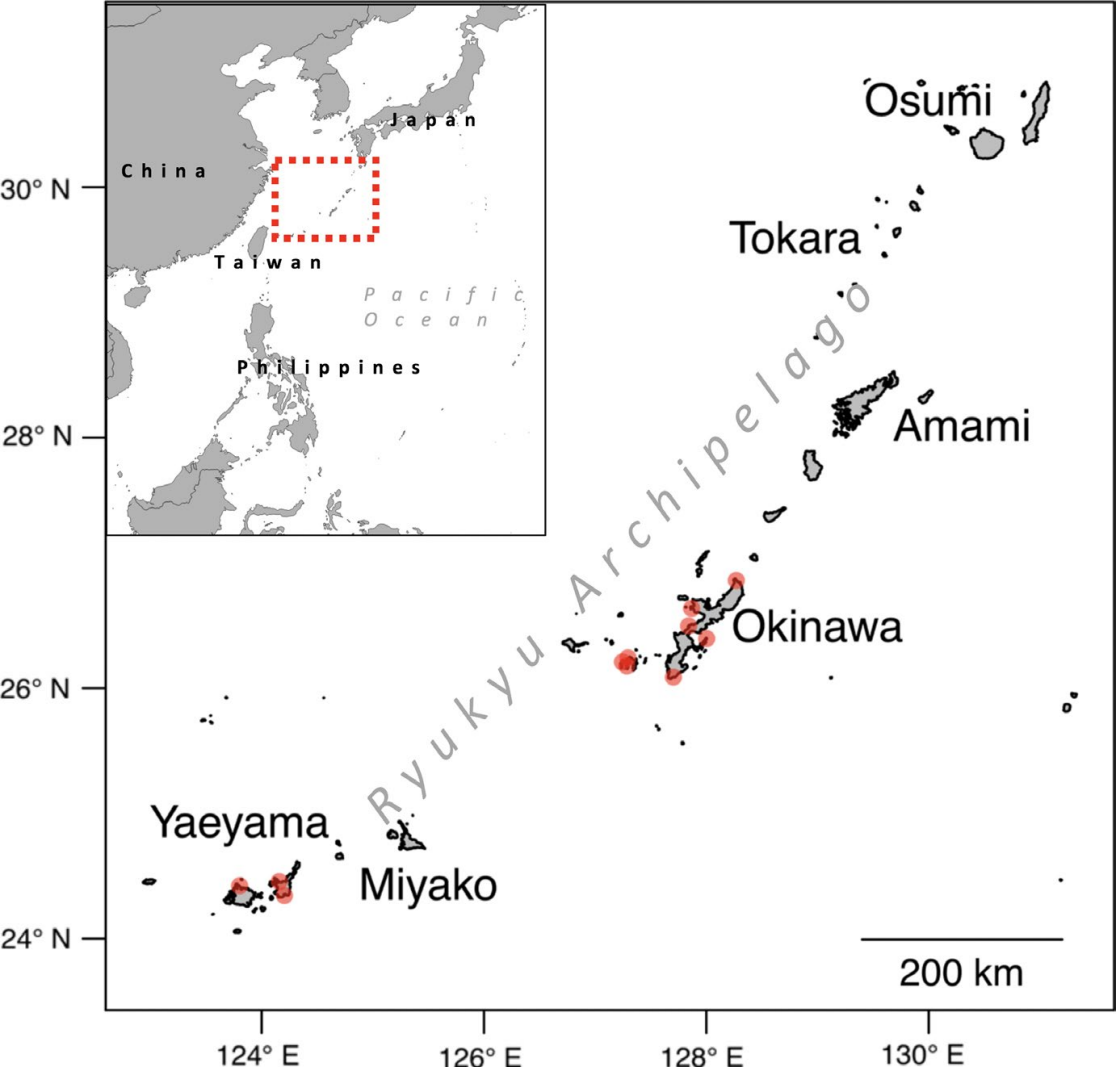
Study area. The Ryukyu Archipelago extends for more than 1,000 km in the north‐western Pacific Ocean. The red circles display the 11 sites where samples were collected for the seascape genomics analysis (adapted from Shinzato et al., 2015)

In the present study, we applied a seascape genomics framework to detect coral reefs that are carrying potentially heat stress adapted genotypes and, in turn, to show how conservation policies could implement the results. Our study focuses on *Acropora digitifera* of the Ryukyu Archipelago in Japan (Figure 1), an emblematic species of the Indo‐Pacific and flagship organism for studies on corals genomics (Shinzato et al., 2011). We first analysed the convergence between genomic and environmental information to (a) detect SNPs potentially conferring a selective advantage and (b) develop a model describing connectivity patterns. Next, we took advantage of these findings to infer which reefs were more likely to be carrying heat stress adapted genotypes and to evaluate their interconnectedness with the rest of the reef system. Finally, we propose an approach to implement the results obtained into conservation planning. Overall, our work provides tools for the interface between conservation genomics and marine environmental sciences, which are likely to empower preservation strategies for coral reefs into the future.

## 2 MATERIALS AND METHODS

**Figure 2:**
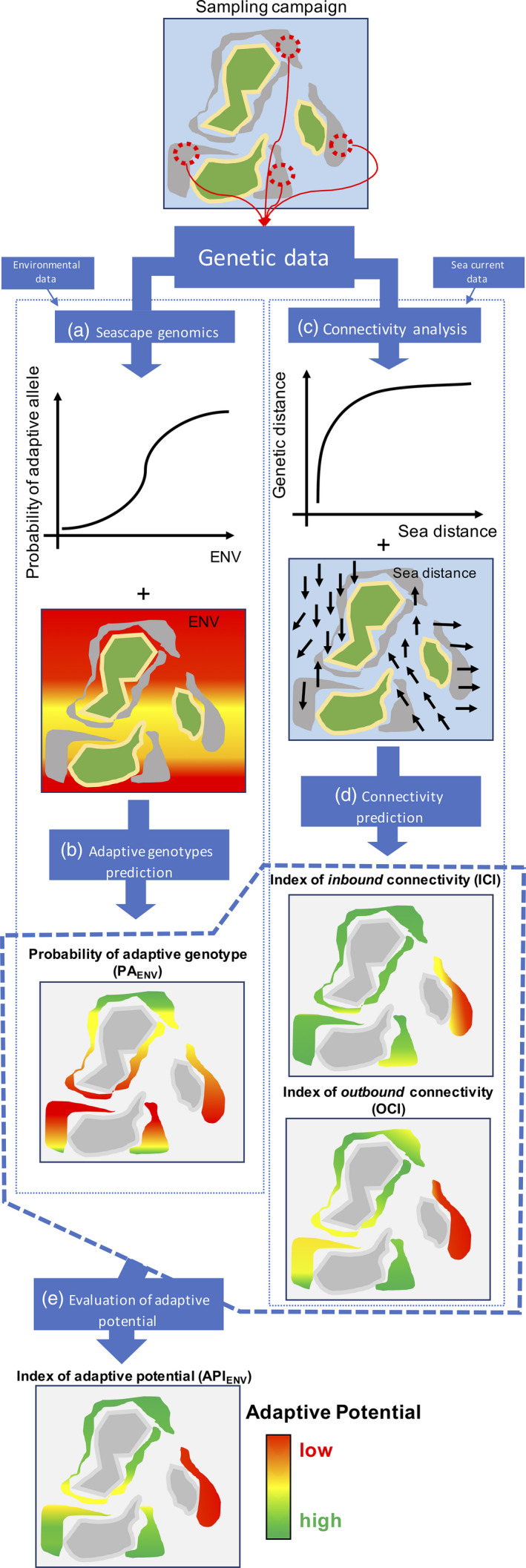
Workflow between the steps of the approach. The starting point for the analysis is the generation of genetic data describing the genotypes observed at different sampling locations (in this example, 4 sampling sites). In the seascape genomics analysis (a), these data are combined with environmental information to uncover genotypes whose frequencies are associated with specific climatic conditions (ENV). Such genotypes are defined as potentially adaptive against the environmental condition of interest. The model describing this link is then applied to environmental data at the scale of the whole reef system (b), to predict the probability of presence of the adaptive genotypes (green: high probability; red: low probability). The genetic data are also combined with sea current information to build a connectivity model (c) describing how sea distances correspond to genetic separation between sampling sites. This model is fitted with sea distance between all the reefs of the study area to predict (d) patterns of connectivity from (outbound) and to (inbound) each reef (green: high connectivity; red: low connectivity). Finally, predictions of the presence of adaptive genotypes and connectivity patterns are combined to assess the adaptive potential across the study area (e): reefs that are connected with sites that are likely to carry the adaptive genotype will have a higher adaptive potential (green), while those that are isolated will have lower adaptive potential (red)

Our framework is structured on two axes of analysis and prediction: one focusing on the presence of putative heat stress adapted genotypes (seascape genomics) and the other on population connectivity (Figure 2). The seascape genomics analysis (Figure 2a) combines genomic data with environmental information to uncover potentially adaptive genotypes at sampling sites. The models describing these relationships are then used to predict, at the scale of the whole study area, the probability of the presence of heat stress adapted genotypes (Figure 2b). In the connectivity study (Figure 2c), we designed a model describing how distances based on sea currents (calculated on the basis of remote sensing data) correspond to the genetic separation between corals at these sites. This model is then used to predict connectivity of sites at the study area scale (Figure 2d). Finally, the predictions of where the heat stress adapted genotypes are more likely to exist, and of how the reef system is interconnected, allow the assessment of adaptive potential across the whole study area (Figure 2e).

### 2.1 Genomic dataset

The genomic data used come from a publicly available dataset consisting of 155 georeferenced colonies of *A. digitifera* from 12 sampling locations (13 ± 5 colonies per site) of the Ryukyu Archipelago in Japan (Figure 1; Bioproject Accession PRJDB4188). These samples were sequenced using a whole‐genome sequencing approach in the scope of a population genomics study. Details on how samples were collected and processed for genomic analysis can be found in Shinzato, Mungpakdee, Arakaki, and Satoh (2015).

Raw genomic data were processed using the Genome Analysis Toolkit framework (GATK; McKenna et al., 2010) following the recommended pipeline (the “GATK Best Practices”; Van der Auwera et al., 2013) with the necessary modifications for coping with the absence of reliable databases of known variants for this species. In short, the *A. digitifera* reference genome (v. 1.1, GenBank accession: GCA_000222465.2; Shinzato et al., 2011) was indexed using bwa (v. 0.7.5a, Li & Durbin, 2009), samtools (v. 1.9, Heng Li et al., 2009) and picard‐tools (v. 1.95, http://broadinstitute.github.io/picard) and raw sequencing reads were aligned using the bwa *mem* algorithm. The resulting alignments were sorted, marked for duplicate reads, modified for read‐group headers and indexed using picard‐tools. Next, each alignment underwent an independent variant discovery using the GATK HaplotypeCaller tool (using the ERC mode and setting the ‐‐minPruning flag to 10) and genotypes were then jointly called by the GATK GenotypeGVCFs tool in random batches of 18 samples to match our computational power (18 CPUs). The variant‐calling matrices of the different batches were then joined and filtered in order to keep only bi‐allelic single nucleotide polymorphisms (SNPs) using the GATK CombineVariants and SelectVariants tools, respectively. This resulted in a raw genotype matrix counting ~ 1.2 M of SNPs. Subsequently, we used the GATK VariantAnnotator tool to annotate variants for quality by depth and filtered for this value (<2), read coverage (>5 and < 100 within a sample), minor allele frequency (>0.05), major genotype frequency (<0.95) and missing rate of both individuals and SNPs (<0.1) using the GATK VariantFiltrationTool and custom scripts in the R environment (v. 3.5.1, R Core Team, 2009, 2016). Finally, we filtered for linkage disequilibrium using the *snpgdsLDpruning* function of the SNPrelate R package (v. 1.16, LD threshold = 0.3; Zheng et al., 2012). This pipeline produced the filtered genotype matrix consisting of 136 individuals and 7,607 SNPs.

Natural hybridization and transient species boundaries have been observed in *Acropora* species (van Oppen, Willis, Rheede, & Miller, 2002) and might cause bias in the analysis of adaptation and connectivity. For this reason, we investigated the presence of these phenomena by running a preliminary analysis of fixation index (F_ST_) variation by genomic position using the R KRIS package (v. 1.1; Chaichoompu et al., 2018). Since we found no genomic islands of low‐recombination (i.e. high F_ST_; Nosil, Funk, & Ortiz‐Barrientos, 2009) when comparing the populations of Kerama, Yaeayama and Okinawa (Figure S1), we excluded the possibility of presence of genetically isolated groups in the data set. Importantly, previous studies on this coral population did not report hybridization with other species, neither the presence of cryptic species nor isolated sub‐populations (Nakajima, Nishikawa, Iguchi, & Sakai, 2010; Nishikawa, 2008; Shinzato et al., 2015).

### 2.2 Environmental data

Seascape genomics analyses require an exhaustive characterization of the environment in order to distinguish the effect of collinear gradients (Leempoel et al., 2017; Rellstab et al., 2015; Riginos et al., 2016). Six georeferenced data sets describing atmospheric and seawater conditions were retrieved from publicly available resources (EU Copernicus Marine Service, 2017; National Oceanic & Atmospheric Administration, 2017; Table S1). All these data sets provided records over several years (on average 15) before the genetic data were sampled (2010; Shinzato et al., 2015), covering the entire study area (Figure 1) with a spatial resolution ranging from ~ 9 to 4 km (Table S1). Four of these data sets (sea surface temperature, salinity, chlorophyll concentration and current velocity) were captured at a daily temporal resolution, while the other two (suspended particulate matter and photosynthetically available radiations) provided monthly averages. We processed these variables in the R environment using the *raster* package (v. 2.8, Hijmans, 2016) to compute for each: (a) the overall average; (b) the highest monthly average and (c) the lowest monthly average. For the four variables captured at a daily temporal resolution, we also computed the standard deviations associated with the three averages.

Furthermore, sea surface temperature measurements were used to compute the bleaching alert frequency (BAF), representing the percentage of days (over the 23 years of remote sensing) during which the heat stress (Liu, Strong, & Skirving, 2003) accumulated over 2 weeks exceeded 4°C. Sea surface temperature and salinity records were combined in a polynomial equation to produce estimates of seawater alkalinity (Lee et al., 2006). Bathymetry data (Ryan et al., 2009) were used to retrieve the depth at sampling locations. Finally, population density data (CIESIN Columbia University, 2010) were averaged in a 50‐km buffer area to produce a surrogate variable for anthropogenic pressure (Welle, Small, Doney, & Azevedo, 2017). In total, 39 environmental variables were computed.

We used the geographic coordinates associated with each sample to characterize the environmental conditions using the QGIS point sampling tool (v. 2.18.25, QGIS development team, 2009, 2016). For the predictive step of our study (Figure 2c) at the scale of the whole reef system, we retrieved the shapes of the reefs of the region (UNEP‐WCMC et al., 2010) and reported them into a regular grid (cell size of 5 × 5 km) using QGIS. For the reef cells smaller than 5 km^2^, we calculated the actual area (in km^2^), as it will be required for the computation of connectivity and adaptive potential indices. Reefs from the neighbouring regions (Taiwan and Philippines, Figure 1) were also included to avoid border effects in computations. Environmental conditions were assigned to each reef cell using the average function of the QGIS zonal statistics tool.

### 2.3 Seascape genomics

The seascape genomics analysis was carried out to investigate the possible correlation between environmental variables and the frequency of particular genotypes. Associations of this kind might reveal an environmental constraint requiring adaptation in *A. digitifera*, as well as the genetic features conferring the selective advantage.

We performed the genotype–environment association analysis using the logistic regression method implemented within the SamBada software (v. 0.7; Duruz et al., 2019; Stucki et al., 2017). The SamBada approach allows proxy variables of genetic structure to be included in the analysis in order to avoid possible confounding effects (patterns of neutral genetic variation mimicking signals of adaptation to the local environment; Holderegger et al., 2008). Here, we performed a discriminant analysis of principal components (DAPC) on the SNPs genotype matrix using the R package *adegenet* (v. 2.1.1; Jombart, 2008). This procedure highlighted a main separation between two groups of samples along the first discriminant function (Figure S2). The latter was therefore used as co‐variable in association models.

The genotype–environment association analysis with SamBada evaluated 890,019 association models (39 environmental variables matched against the 3 genotypes of the 7,607 bi‐allelic SNPs). For each association model related to the same environmental variable, *p*‐values of *G*‐scores (*G*) and Wald scores (*W*) were corrected for multiple testing using the R *q‐value* package (v. 2.14, Storey, 2003). Association models scoring *q* < 0.01 for both statistics were deemed significant. If a SNP was found in more than one significant association (e.g. with collinear environmental variables), only the best model (according to the value of *G*) was kept. This best association model is hereafter referred to as the significant genotype–environment association (SGEA).

### 2.4 Annotation of seascape genomics results

Since landscape/seascape genomics analysis can suffer the issue of false positives, it is important to use a complementary method to strengthen SGEAs (Rellstab et al., 2015). In this work, we annotated the genomic neighbourhood of each SGEAs and verified whether the molecular functions of the genes surrounding a SNP were coherent with a presumptive adaptive role.

We set the maximum size of the search window to ± 250 kbs around the concerned SNP of each SGEA. This maximal window size was selected because genes(s) possibly linked to a mutation may lay up to hundreds of kbs away (Brodie, Azaria, & Ofran, 2016; Visel, Rubin, & Pennacchio, 2009), and this window size corresponds approximately to the scaffold N50 statistics of the reference genome (i.e*.* half of the genome is contained within scaffolds of this size or longer).

For every SGEA, the annotation procedure was performed as follows. Based on the NCBI annotation of the reference genome (https://www.ncbi.nlm.nih.gov/genome/annotation_euk/Acropora_digitifera/100/), we retrieved all the predicted genes falling within the ± 250 kbs window. Next, we retrieved the predicted protein sequences related to these genes and ran a similarity search (blastp, (Madden & Coulouris, 2008) against metazoan protein sequences in the swissprot database (release 2019_07; Boeckmann et al., 2003). For every predicted gene, only the best significant match (E‐score threshold < 10^–7^) was kept. Finally, predicted genes were annotated with the eukaryotic cluster of orthologous genes (KOG; Jensen et al., 2008) annotation from the matching swissprot entry. For every KOG, we calculated the relative frequency across the *A. digitifera* genome. This was obtained by dividing the genome into 500 kbs windows and by calculating the percentage of windows in which the KOG was observed.

### 2.5 Probability of presence of heat stress adapted genotypes

The seascape genomics analysis pointed out genotypes expected to confer a selective advantage under a determined environmental condition. Furthermore, the SamBada approach provided, for every SGEAs, the parameters of a logistic regression that links the probability of occurrence of the adaptive genotype with the value of the environmental variable (Figure S3; Stucki et al., 2017). These logistic models can therefore be used to estimate the probability of presence of the genetic variant for any value of the environmental variable at any reef of the Ryukyu Archipelago (Joost, 2006; Rochat & Joost, 2019).

For SGEAs related to a same environmental pressure, these single genotype probabilities can be combined into an average probability (i.e. the arithmetical mean) of carrying genotypes adapted to a specific condition ( ${\text{PA}}_{\text{env}}$ ). In this study, we applied this calculation to a group of SGEAs related to heat stress (high bleaching alert frequency) that showed functional annotations coherent with a role in heat response (SGEA3, 5–8 and 13; Table 1). The resulting value was the probability of carrying heat stress adapted genotypes ( ${\text{PA}}_{\text{heat}}$ ).

**Table 1:**
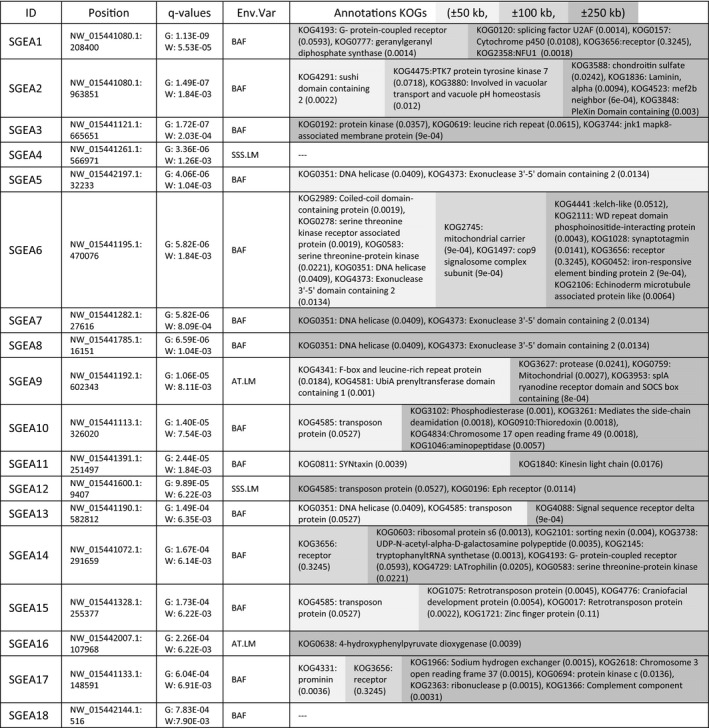
Significant genotype–environment associations (SGEA). The seascape genomics analysis using the SamBada method detected 18 significant (qG and qW < 0.01) genotype–environment associations (SGEA). This table shows, for each SGEA, the genomic position of the concerned SNP (in the format scaffoldID:position; Position), the q‐values related to the G‐score (G) and the Wald score (W) of the association model, the concerned environmental variables (BAF: bleaching alert frequency, SSS.LM: lowest average monthly salinity, AT.LM: lowest average monthly alkalinity; Env. Var.), the eukaryote cluster of orthologous genes (KOGs) annotated within ± 50 kb (light grey), ± 100 kb (grey) and ± 250 kb (dark grey) around the concerned SNP. For every KOG annotation, the frequency of the term across the reference genome is given in brackets

### 2.6 Sea current data

The starting point for the connectivity analysis and prediction was the evaluation of how pairs of reefs are expected to be connected by water flow. This step was carried out by processing remote sensing data on water current to construct a matrix that defines the costs of transitions from one reef to another.

Daily records of sea surface current were retrieved from publicly available databases (zonal and meridional surface velocities from the *global‐reanalysis‐phy‐001‐030* product; EU Copernicus Marine Service, 2017) and used to compute the direction and speed of currents in the R environment using the *raster* library. By using the resample function of the R *raster* library, we downscaled these data from original 0.083° (~9.2 km) to 0.015° (~1.6 km) and corrected land pixels (i.e. removing sea current values) using a high‐resolution bathymetry map (Ryan et al., 2009). These day‐by‐day records of sea currents (from 1993 to 2010) were then stacked to retrieve, for each pixel of the study area, the cumulative speed towards each of the eight neighbouring pixels. For every pixel, the cumulative speed in each of the eight directions was divided by the total speed (the sum of the eight directions) to obtain the probability of transition in each direction (the conductance). This information was used to calculate dispersal costs (the inverse of the square conductance) and was summarized in a transition matrix in the format of the R *gdistance* package (v. 1.2, van Etten, 2018).

For the connectivity analysis (Figure 2c), the transition matrix was used to calculate sea distances (i.e. the least‐cost path) between sampling sites of the genotyped colonies. For the connectivity predictions (Figure 2d), we calculated the sea distances between all the reefs of the study area (the 5 × 5 km cells described in the environmental variables section). Importantly, for each pair of reefs (for instance reef_1_ and reef_2_) two sea distances were computed, one for each direction (i.e. from reef_1_ to reef_2_ and from reef_2_ to reef_1_). The result of this calculation was an asymmetrical square matrix describing sea distance between any reef cell.

### 2.7 Connectivity analysis

The connectivity analysis was performed to estimate how a unit of sea distance between two reefs is translated in terms of genetic separation between *A. digitifera* colonies. This step is necessary because sea distance does not account for the biological differences (for instance differential larval survival period) between different species.

Genetic distances between sampling sites were calculated using the pairwise F‐statistics (F_ST_; Weir & Cockerham, 1984) as implemented in the R *hierfstat* library (v. 0.04; Goudet, 2005). When there is no dispersal constraint between two sub‐populations, the related F_ST_ is equal to zero. Conversely, when dispersal is constrained, F_ST_ increases up to a maximum value of one (isolated sub‐populations). To avoid bias due to low sample sizes, we only considered sample sites with more than 10 samples each (7 out of 12).

Next, we built a linear model (hereafter referred to as the connectivity model) to estimate F_ST_ from the shortest sea distance (least‐cost path) between each pair of sample sites. As a comparison, we built a connectivity model using Euclidean distances of coordinates (aerial distances) as independent variable while maintaining F_ST_ as response variable. The quality of models was estimated by calculating the coefficients of determination (R^2^) and the Akaike information criterion (AIC; Bozdogan, 1987).

### 2.8 Connectivity predictions

**Figure 3:**
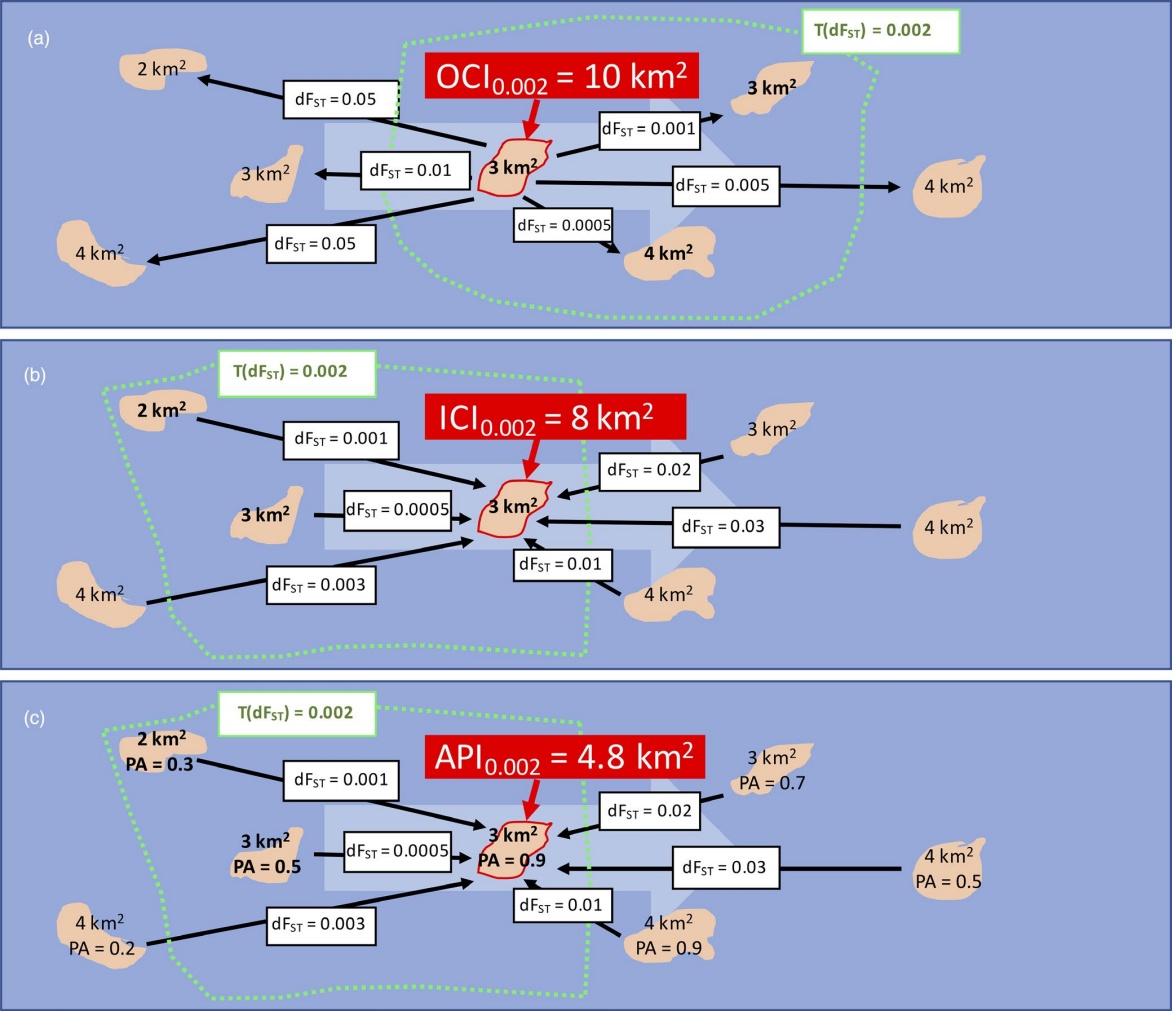
Calculation of connectivity and adaptive potential indices. The three maps display a hypothetical seascape with seven reefs (in rose) of different extent and connected by sea current flowing from left to right (large light blue arrow). On each map, a different index is calculated for the same focal reef (highlighted in red): (a) outbound connectivity index (OCI), (b) inbound connectivity index (ICI) and (c) adaptive potential index (API). The black arrows display the estimated directional genetic separation (dF_ST_) for corals travelling from (a) and towards (b, c) the focal reef. The calculation of the indices requires that a threshold value for dFst is set (in this example, T(dFst)=0.002, the green border) in order to define the reefs neighbouring the focal one. OCI (a) represents the total area (in km^2^) of neighbouring reefs (destinations) that can be reached from the focal reef (departure). ICI (b) represents the total area of neighbouring reefs (departures) that can reach the focal reef (destination). API (c) is a special case of ICI, where the area of the neighbouring reefs is weighted by their probability of presence of adapted genotypes (PA)

The model that was developed during the connectivity analysis describes how a unit of sea distance is translated into a unit of genetic separation (F_ST_) in *A. digitifera* (Figure S4). Since we previously characterized the sea distances between any reef of the Ryukyu Archipelago, here we translated such physical distances into predicted degrees of genetic separation. This transformation was applied to the asymmetrical square matrix describing sea distances between any reef cell of the study area. The resulting matrix contains the corresponding directional estimates of genetic separation (dFst; Figure S5) and is employed to calculate two indices that summarize connectivity for every reef cell:

- *outbound connectivity index* (OCI; Figure 3a): OCI describes how a specific reef (departure reef) is expected to disperse towards neighbouring reefs (destination reefs). More specifically, OCI represents the total area (in km^2^) of neighbouring destination reefs that can be reached from the departure reef within a determined dF_ST_ distance.
- *inbound connectivity index* (ICI; Figure 3b): ICI describes how a specific reef (destination reef) is expected to receive recruits from neighbouring reefs (departure reefs). More specifically, ICI represents the total area (in km^2^) of neighbouring departure reefs that can reach the destination reef within a determined dF_ST_ distance.

These connectivity indices and their interpretation are subordinate to the dF_ST_ threshold applied in the calculation. For this reason, it is crucial to set this threshold by considering the size of the study area and the distribution of the dF_ST_ values observed (Figure S5). In this work, we set the dF_ST_ threshold to 0.02. In fact, a smaller dF_ST_ (for instance 0.01; Figure S5) would have informed on local connectivity only (within neighbouring islands) and neglect connectivity at the scale of the Ryukyu Archipelago. In contrast, a higher dF_ST_ (for instance 0.05, Figure S5) would have exceeded the study area boundaries, causing bias (border effects) in the calculation of the indices for reefs of the southern Islands (Yaeyama and Miyako) of the Archipelago.

### 2.9 Evaluation of the adaptive potential against heat stress

The adaptive potential against heat stress was evaluated by combining the predictions of the presence of heat stress adapted genotypes ( ${\text{PA}}_{\text{heat}}$ ) and connectivity patterns (ICI) in an *index of adaptive potential* against heat stress ( ${\text{API}}_{\text{heat}}$ , Figure 2e). Indeed, ${\text{API}}_{\text{heat}}$ is a special case of ICI calculated as the sum of the weighted area (in km^2^) of all the reefs connected under a specific dF_ST_ threshold to the focal reef (Figure 3c). The weight applied to each reef corresponded to the probability of carrying heat stress adapted genotypes ( ${\text{PA}}_{\text{heat}}$ ). For the dF_ST_ threshold, we used the same value (0.02) as employed in the ICI and OCI calculations.

## 3 RESULTS

### 3.1 Seascape genomics

We detected 18 significant genotype–environment associations (SGEA, q_G_ and q_W_ < 0.01, Table 1) spanning across 17 distinct scaffolds of the *A. digitifera* reference genome. Among them, 14 were related to bleaching alert frequency (BAF), two to lowest average monthly salinity (SSS) and two to lowest monthly average alkalinity (AT).

The functional annotations surrounding SNPs involved in SGEAs showed that in nine cases the closest genes belonging to eukaryotic clusters of orthologs (KOGs) fell within a ± 50 kb window, in two within ± 100 kb, in five within ± 250 kb and in two over ± 250 kb (Table 1). In total, 64 KOGs were annotated and some recurred in SNPs from different SGEAs, such as *DNA helicases* (in SGEA5‐8 and 13, all related to BAF), *transposon protein* (SGEA10, 12, 13 and 15), *exonuclease 3'‐5' domain containing* (SGEA5‐8, all related to BAF), *serine–threonine protein kinase* (SGEA6 and 14, both related to BAF) and *G protein‐coupled receptor* (SGEA1 and 14, both related to BAF). The remaining KOGs were observed only once, and among those expected at lowest frequency (<0.001 per ± 250 kb window) across the *A. digitifera* genome, we found *jnk1 mapk8* *‐associated membrane protein* (SGEA3, associated with BAF), *mitochondrial carrier and iron‐responsive element binding protein 2* (SGEA5, associated with BAF), *splA ryanodine receptor domain* (SGEA9, associated with AT) and *signal sequence receptor delta* (SGEA13, associated with BAF).

## 4 PROBABILITY OF PRESENCE OF HEAT STRESS ADAPTED GENOTYPES

The SGEAs of the seascape genomics analysis were then used as the starting point for predicting the probability of presence of heat stress adapted genotypes ( ${\text{PA}}_{\text{heat}}$ ) across the reefs of the region. For the calculation of this probability, we employed six SGEAs (SGEA3, 5–8 and 13) related to bleaching alert frequency that displayed functional annotations coherent with a role in heat stress resistance (Table 1).

**Figure 4:**
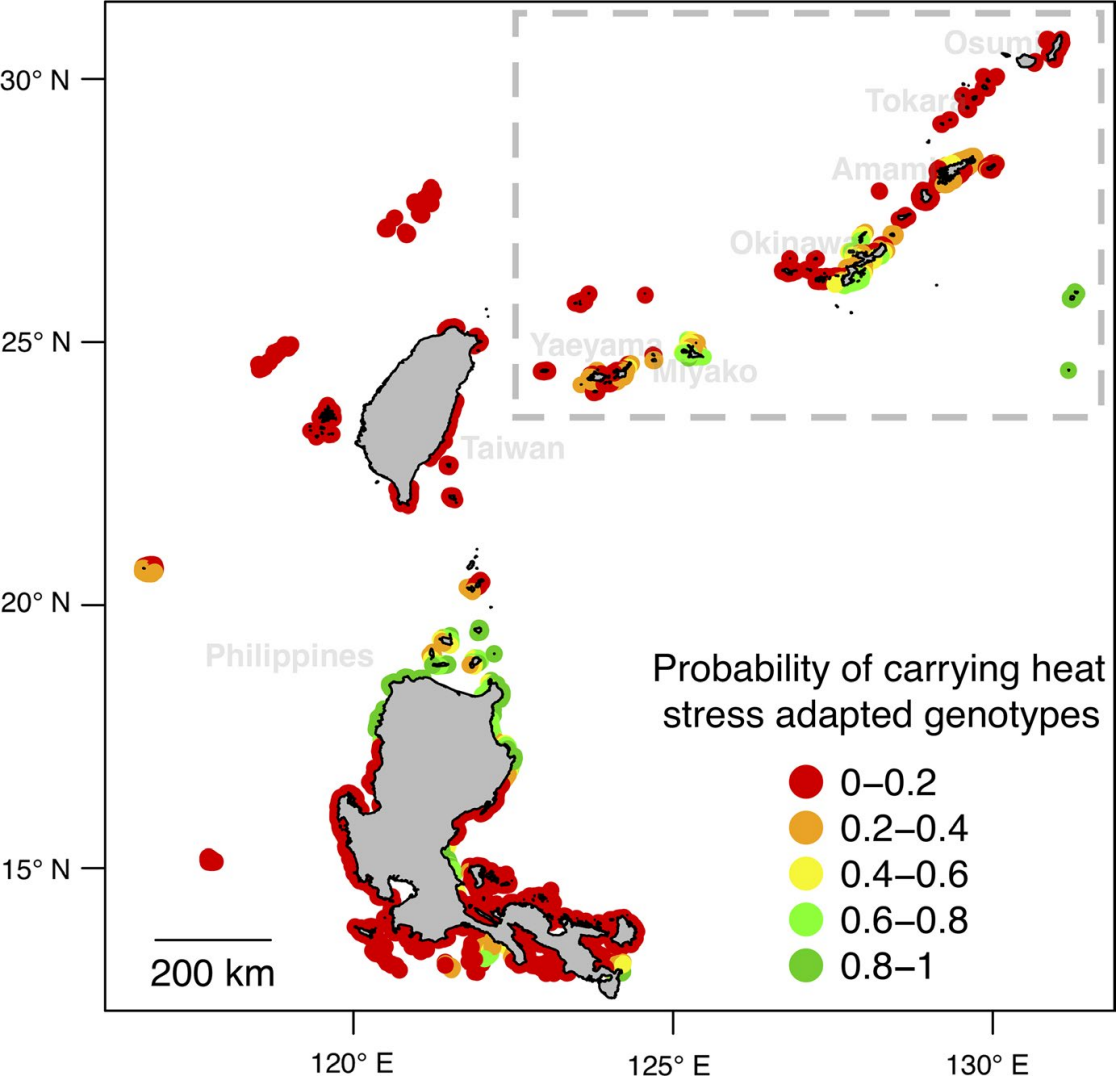
Probability of carrying heat stress adapted genotypes ( ${\text{PA}}_{\text{heat}}$ ). The map shows the probability of presence of the genotypes expected to be linked to adaptation against heat stress across the study area and the neighbouring regions. Seven significant gene–environment associations (SGEA1, 3, 5–8 and 13, Table 1) describing the association between distinct genotypes and bleaching alert frequency were used to predict expected genotype frequencies. These expected frequencies were then averaged to compute the cumulated probability of adaptive genotypes. The dashed box highlights the position of the Ryukyu Archipelago

The average of ${\text{PA}}_{\text{heat}}$ ranged from 0 to 1 (Figure 4). In Ryukyu Archipelago, ${\text{PA}}_{\text{heat}}$ was higher in Miyako ( ${\bar{\text{PA}}}_{\text{heat}\;\text{Miyako}}=0.47\pm 0.21$ ) and Okinawa ( ${\bar{\text{PA}}}_{\text{heat}\;\text{Okinawa}}=0.33\pm 0.21$ ), lower in Amami ( ${\bar{\text{PA}}}_{\text{heat}\;\text{Amami}}=0.18\pm 0.12$ ) and Yaeyama ( ${\bar{\text{PA}}}_{\text{heat}\;\text{Yaeyama}}=0.18\pm 0.09$ ) and close to zero in the north of the region (Tokara and Osumi; ${\bar{\text{PA}}}_{\text{heat}\;\text{Tokara}}=0.02\pm 0.03$ , ${\bar{\text{PA}}}_{\text{heat}\;\text{Osumi}}=∼0$ ; Figure 4). Outside the Ryukyu Archipelago, a high ${\text{PA}}_{\text{heat}}$ (>0.8) was predicted in northern Philippines while reefs around Taiwan displayed in general low ${\text{PA}}_{\text{heat}}$ (<0.2; Figure 4).

### 4.1 Connectivity modelling

The connectivity model used for the calculation of the connectivity indices accounted for 72% of the F_ST_ variation (R^2^ = 0.72, AIC=−234; Figure S4a) and resulted as a more accurate model when compared to the one based on aerial distance (R^2^ = 0.66, AIC=−230, Figure S4b).

**Figure 5:**
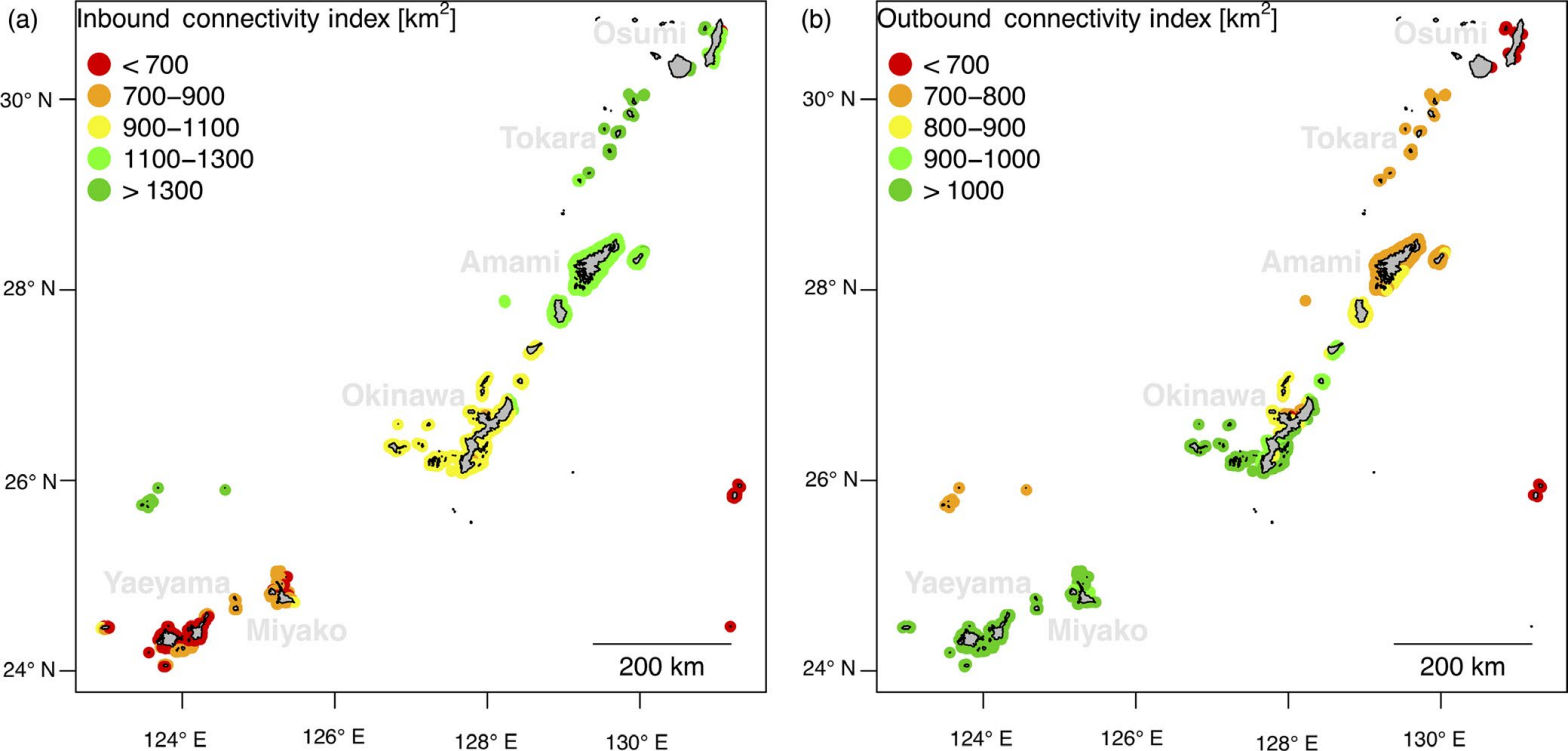
Connectivity indices. The maps show the potential connectivity to (a) and from (b) every reef of the Ryukyu Archipelago. In (a), the inbound connectivity index (ICI) represents the total area (in km^2^) of the reefs that are connected to the focal reef with a dF_ST_ < 0.02 (dF_ST_ towards the focal reef). Reefs with a high ICI are expected to receive recruits from a larger neighbourhood. In (b), the outbound connectivity index (OCI) displays the total area of the reefs that are connected from the focal reef with a dF_ST_ < 0.02 (dF_ST_ from the focal reef). Reefs with a high OCI are expected to disperse towards a larger neighbourhood

The ICI variation followed a north to south decrease (Figure 5a). The reefs around the islands in the north of the archipelago (Osumi, Tokara and Amami) were generally those with the highest ICI ( ${\bar{\text{ICI}}}_{\text{Tokara}}=1615\pm 229{\text{km}}^{2}$ ; ${\bar{\text{ICI}}}_{\text{Amami}}=1209\pm 28{\text{km}}^{2}$ ; ${\bar{\text{ICI}}}_{\text{Osumi}}=1164\pm 336$ km^2^; Figure 5a). In the central area (Okinawa), ICI was lower ( ${\bar{\text{ICI}}}_{\text{Okinawa}}=999\pm 42{\text{km}}^{2}$ ), while the lowest ICI values were observed in the southern area (Yaeyama and Miyako; ${\bar{\text{ICI}}}_{\text{Miyako}}=777\pm 71{\text{km}}^{2};{\bar{\text{ICI}}}_{\text{Yaeyama}}=674\pm 76{\;\text{km}}^{2}$ ; Figure 5a).

With regard to OCI, we observed a decrease in index with increasing latitude (Figure 5b). OCI was highest in the southern half of the archipelago (Yaeyama, Miyako and Okinawa; ${\bar{\text{OCI}}}_{\text{Yaeyama}}=1014\pm 2{\text{km}}^{2}$ ; ${\bar{\text{OCI}}}_{\text{Miyako}}=1008\pm 14{\text{km}}^{2}$ ; ${\bar{\text{OCI}}}_{\text{Okinawa}}=936\pm 91\;{\text{km}}^{2}$ ; Figure 5b). A lower OCI was observed in the northern part (Amami and Tokara; ${\bar{\text{OCI}}}_{\text{Amami}}=766\pm 51{\text{km}}^{2}$ ; ${\bar{\text{OCI}}}_{\text{Tokara}}=706\pm 2{\text{km}}^{2}$ ; Figure 5b), while the extreme north of the Archipelago (Osumi) had a very low OCI ( ${\bar{\text{OCI}}}_{\text{Osumi}}=6\pm 4{\text{km}}^{2}$ ; Figure 5b).

### 4.2 Evaluation of the adaptive potential

**Figure 6:**
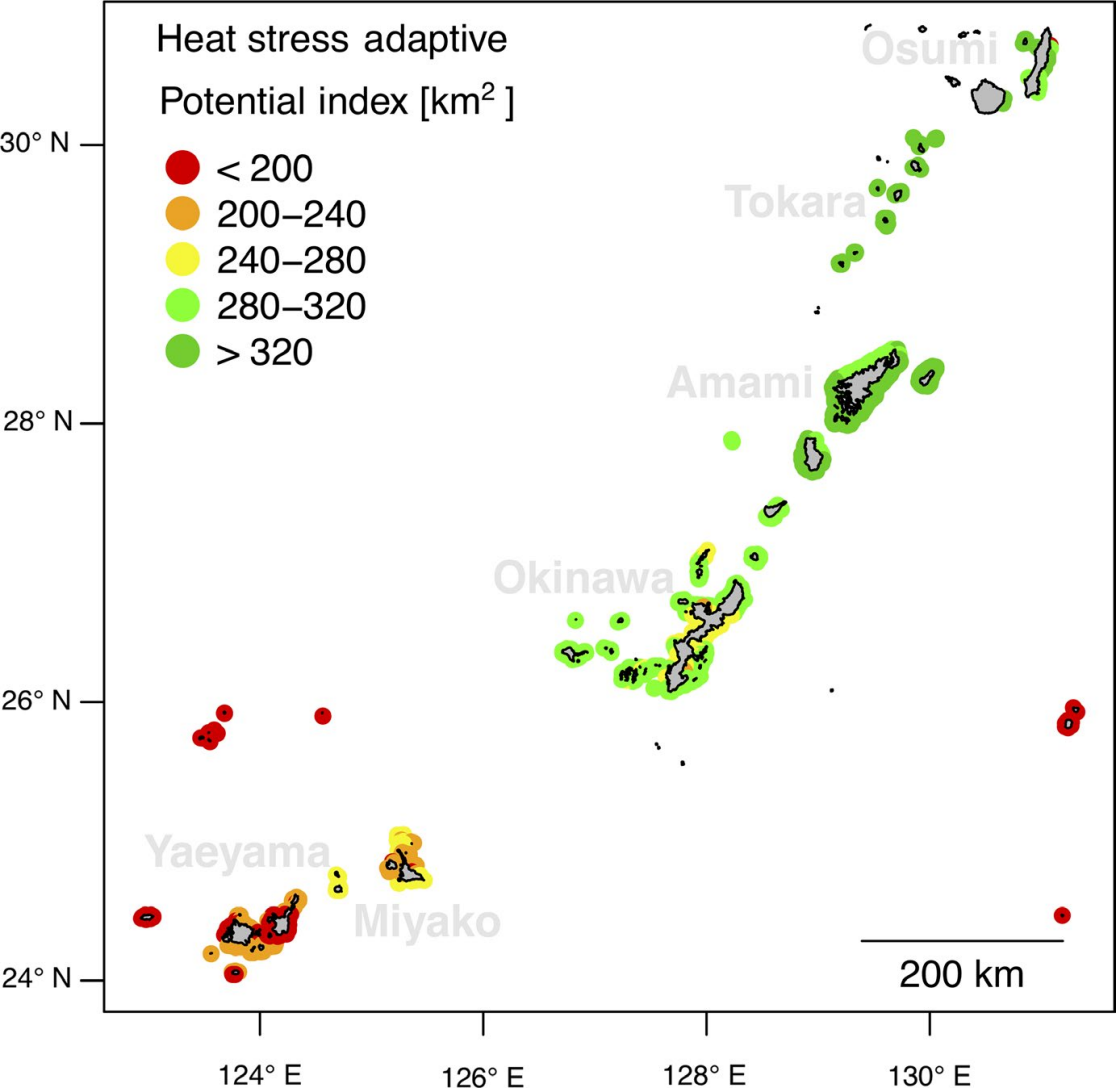
Index of adaptive potential against heat stress ( ${\text{API}}_{\text{heat}}$ ). The map displays the index of adaptive potential against heat stress (high bleaching alert frequency, BAF) for every reef of the study area. This index represents the sum of weighted areas of reefs connected to the focal reef with a pFst < 0.02 (pFst towards the focal reef). The weight applied corresponds to the probability of carrying heat stress adapted genotypes ( ${\text{PA}}_{\text{heat}}$ ). Reefs with a large API are expected to receive more heat stress adapted recruits

The variations of ${\text{API}}_{\text{heat}}$ were generally structured along the latitudinal axis (Figure 6). Reefs in the northern part of the Archipelago (Tokara, Amami and Osumi) generally showed the highest ${\text{API}}_{\text{heat}}$ values ( ${\bar{\text{API}}}_{{\text{heat}}_{\text{Tokara}}}=335\pm 6{\text{km}}^{2}$ ; ${\bar{\text{API}}}_{{\text{heat}}_{\text{Amami}}}=317\pm 10{\text{km}}^{2};{\bar{\text{API}}}_{{\text{heat}}_{\text{Osumi}}}=296\pm 86{\text{km}}^{2}$ ; Figure 6). In the central part of the study area (Okinawa), ${\text{API}}_{\text{heat}}$ was lower ( ${\bar{\text{API}}}_{{\text{heat}}_{\text{Okinawa}}}=279\pm 12{\text{km}}^{2}$ ; Figure 6), and in the southern part, the lowest ${\text{API}}_{\text{heat}}$ values were observed ( ${\bar{\text{API}}}_{{\text{heat}}_{\text{Yaeyama}}}=200\pm 17{\text{km}}^{2}$ ; ${\bar{\text{API}}}_{{\text{heat}}_{\text{Miyako}}}$ $=237\pm 24{\text{km}}^{2}$ ; Figure 6).

## 5 DISCUSSION

### 5.1 Adaptation to heat stress

Heat stress is expected to be one of the major threats to coral reef survival, where the research for adaptive traits is becoming of paramount importance (Baums, 2008; Logan et al., 2014; Maina et al., 2011). In the present study, the seascape genomics analysis of *A. digitifera* of the Ryukyu Archipelago revealed the presence of 14 genomic regions hosting genetic variants that might confer a selective advantage against heat stress (Table 1). None of the SNPs related to the SGEA lay directly within a coding sequence of a putative gene, but this is rarely the case for causative mutations (Brodie et al., 2016). In fact, genetic variants in intergenic regions that play a modulatory action on the expression of neighbouring genes are more frequent and can influence loci at a distance of 1–2 Mb (Visel et al., 2009). The fragmentation of the reference genome forced us to limit our search window to ± 250 Kb around each SNP, yet we still found annotations corroborating a response to heat stress.

The SNP in SGEA3 was found to be related to KOG3744 (*jnk1 mapk8* *‐associated membrane protein;* Table 1). This KOG is rare across the genome of *A. digitifera* (with an expected frequency of 0.0009 per 500 kbs window), and previous research corroborates the hypothesis that this gene plays a role in thermal adaptation. In fact, mitogen‐activated protein kinases (MAPKs) are proteins known to be involved in cellular responses to stress across a range of taxa (Neupane, Nepal, Benson, MacArthur, & Piya, 2013), and the c‐Jun‐N‐terminal kinase (JNK) has previously been shown to be activated under thermal stress in the coral *Stylopohora pistillata* (Courtial et al., 2017).

In SGEA3‐8 and 13, one KOG recurred in the annotations: KOG0351 (*DNA helicase*; Table 1). The expected frequency of this KOG is 0.04 per 500 kbs window, and remarkably, we found five of them in five distinct 500 kbs windows around SGEA associated with heat stress. Of note, in these 5 SGEAs *DNA helicase* was consistently the closest KOGs annotated around the SNPs concerned (Table 1). KOG0351 annotates a particular type of DNA helicases (swissprot IDs: Q91920, Q14191) known as “helicases Q” or “RecQ” (Box S1, 5–7), which are involved in the DNA repairing mechanism caused by UV‐light damage in prokaryotes (Courcelle & Hanawalt, 1999), and for which light‐stress driven effects were observed in eukaryotic cells as well (Sharma, Doherty, Brosh, & Jr., 2006). The modulation of this mechanism might therefore play a role in increasing *A. digitifera* resistance against light‐stress associated with heatwaves.

### 5.2 Connectivity patterns

Coral dispersal is driven by water flow (Paris‐Limouzy, 2011), which is highly asymmetrical in this region (north‐east oriented) due to the Kuroshio Current (Nishikawa, 2008). As previously observed, the main patterns of migrations in this population occurs from the south‐west to the north‐east (Shinzato et al., 2015). Reefs in the southern part of the study area (Yaeyama and Miyako) showed the lowest ICI values (Figure 5a), suggesting a potential lack of recruits arriving from other reefs of the region. In fact, the genetic diversity of southern reefs of the Ryukyu Archipelago is likely to depend on the recruits arriving from the east coast of Taiwan and the northern Philippines, which are located upstream of the Kuroshio Current (Figure S5a; Chen & Shashank, 2009).

In the previous study on this data (Shinzato et al., 2015), reefs from Yaeyama resulted as those with the lowest heterozygosity rates across the study area. This observation was attributed to a population bottleneck caused by the 1998 bleaching event, but it is worth noting that reefs on the west coast of Okinawa showed higher heterozygosity rates despite having suffered recurrent bleaching events since 1998 (Donner, Rickbeil, & Heron, 2017). The lower heterozygosity rates in Yaeyama therefore might reflect not only the effects of past bleaching, but also the relative isolation of these islands from the reefs of the region (Figure 5a).

In line with the same previous observations (Shinzato et al., 2015), the OCI value showed (Figure 5b) that the southern reefs (Yaeyama and Miyako) are those expected to be the most prominent source of recruits for the rest of the Archipelago. Given this crucial aspect, it is even more important to preserve southern reefs of the Ryukyu Archipelago from the risks of isolation (e.g. inbreeding depression; Keller & Waller, 2002).

### 5.3 Heat stress adaptive potential in the 2016 bleaching event

Reefs in islands of Miyako, Okinawa, were those most likely to carry heat stress adapted genotypes (Figure 4). Previous work reported severe bleaching in Okinawa in 1998 (Yamazato, 1999) and that adapted colonies might have resisted (Van Woesik, Irikawa, & Loya, 2004). In contrast, reefs in the northern part of the Archipelago (Amami, Tokara and Osumi) experienced bleaching with moderate severity during the 1998 event (Donner et al., 2017), which might explain why heat stress adapted genotypes are not expected at the same frequency (Figure 4).

The heat stress adaptive potential index ( ${\text{API}}_{\text{heat}}$ ) defines the convergence between the probability of carrying heat stress adapted genotypes with connectivity predictions (Figure 6). Reefs in the northern part of the Archipelago (Amami, Tokara and Osumi) showed a higher ${\text{API}}_{\text{heat}}$ compared to those in the southern half of the region (Okinawa, Yaeyama and Miyako). Two reasons may explain this result: (a) these northern reefs are located downstream (on the Kuroshio Current) of two areas where putative adapted reefs are frequent (Okinawa and Miyako; Figure 4); (b) the region of Northern Philippines, hosting high density of putative adapted reefs (Figure 4), is more connected to the northern part of the Ryukyu Archipelago than with the southern part (Figure S6). This may also explain why, despite hosting putative heat stress adapted reefs (Figure 3), the Miyako area showed among the lowest ${\text{API}}_{\text{heat}}$ values of the Archipelago (Figure 6).

In 2016, the first mass bleaching event occurred in Japan since Shinzato and colleagues published the genetic data re‐analysed in this work (Kimura, Tun, & Chou, 2018). Field surveys related to this bleaching event reported severe bleaching in Yaeyama (intensity up to 99%, mortality up to 68%) and in Miyako (intensity up to 70%, mortality up to 67%; Table 2). In Okinawa and Amami, the impact of this same bleaching event was moderate to mild (Okinawa: intensity up to 48%, mortality up to 13%; Amami: intensity 8% and mortality 2%; Table 2). Reefs predicted with low ${\text{API}}_{\text{heat}}$ (the southern reefs) appeared to suffer more severe bleaching than those in the northern region (which showed higher ${\text{API}}_{\text{heat}}$ ; Figure 6), but care must be taken in the interpretation due to the confounding role of sea temperature during 2016 (Table 2). Indeed, satellite records of sea temperature (EU Copernicus Marine Service, 2017) show that in 2016 the number of days under bleaching alert was higher in the southern part of the Archipelago (Yaeyama: ~84 days; Miyako: ~87 days) than in the northern region (Okinawa: ~76 days; Amami: ~66; Table 2). Nevertheless, when two sites had a comparable degree of heat stress, higher ${\text{API}}_{\text{heat}}$ was generally associated with a reduced severity in bleaching. For instance, reefs in Kerama (Okinawa) and Ishigaki Island West (Yaeyama) suffered 80 and 83 days under bleaching alert in 2016, respectively, but the bleaching intensity in the Ishigaki Island was more than nine times higher than observed for Kerama (63% versus 7%), with a lower ${\text{API}}_{\text{heat}}$ (193 km^2^ versus 282 km^2^; Table 2). Similarly, despite spending 87 days under bleaching alert, reefs in Miyako ( ${\text{API}}_{\text{heat}}$ ~240 km^2^) showed lower bleaching intensity (70%) compared to those in the Sekisei Lagoon (Yaeyama, >95% bleaching) that were predicted with lower ${\text{API}}_{\text{heat}}$ (~200 km^2^).

**Table 2:** Field report of the 2016 mass bleaching event. The table shows the severity and mortality associated with the 2016 bleaching event as reported by Global Coral Reef Monitoring Network (Kimura et al., 2018). For every region surveyed in this report (identified by an ID and a region name), we show the corresponding region in our study and the associated average API against heat stress ( ${\text{API}}_{\text{heat}}$ ), the probability of presence of heat stress adapted genotypes ( ${\text{PA}}_{\text{heat}}$ ) and degree of heat stress in 2016 (estimated as the number of days under bleaching alert). Colour scales highlight the variation of the value of each variable

ID	Region Name	Region Area (this study)	Bleaching [%]	Morality [%]	API_heat_ [km2]	PA_heat_	Bleaching alert [# of days]
3	Amami Islands	Amami	8.5	2.1	318	0.21	66
4	Okinawa Island, East coast	Okinawa	21	0.7	286	0.52	74
5	Okinawa Island, West coast	Okinawa	13.1	4.3	276	0.30	78
6	Okinawa Outer Islands	Okinawa	48.4	13.5	283	0.60	78
7	Kerama Islands	Okinawa	7.3	5.4	282	0.07	80
9	Miyako Island	Miyako	68.8	31	239	0.52	87
10	Miyako Outer Reefs	Miyako	70.1	67.5	248	0.60	87
11	Ishigaki Island, East coast	Yaeyama	47.9	8.8	198	0.30	84
12	Ishigaki Island, West coast	Yaeyama	63.2	14.8	193	0.09	83
13	Sekisei Lagoon, North	Yaeyama	91.5	46.9	192	0.13	84
14	Sekisei Lagoon, East	Yaeyama	99.5	67.9	204	0.23	84
15	Sekisei Lagoon, Center	Yaeyama	94.9	49.7	206	0.19	84
16	Sekisei Lagoon, South	Yaeyama	98.2	50	218	0.16	84
17	Iriomote Islands	Yaeyama	94.3	34.8	202	0.23	84

While these field observations seem to corroborate our predictions on adaptive potential, it is important to consider that they do not refer specifically to *A. digitifera,* but to the coral community as a whole (Kimura et al., 2018). Additionally, other local stressors (for instance anthropogenic pollution) might have modulated the bleaching response (Ateweberhan et al., 2013). Future bleaching surveys, with larger sample sizes and bleaching data referring to the specific coral genus, might provide a more reliable ground for validating our predictions.

### 5.4 Limitations and future directions

Seascape/landscape genomics studies are susceptible to high false discovery rates, especially when the cofounding role of neutral genetic variation is not accounted for (Rellstab et al., 2015). We coped with this issue by running seascape genomics models explicitly integrating demographic processes (Stucki et al., 2017). However, a sampling scheme adapted to seascape genomics (unlike the one used by Shinzato et al., 2015 who did not consider environmental variability) would have further increased sensitivity and lowered false discoveries (Riginos et al., 2016; Selmoni, Vajana, Guillaume, Rochat, & Joost, 2020). In an ideal situation, significant genotype–environment associations should be validated by running experimental assays such common garden or aquaria experiments (Krueger et al., 2017), reciprocal transplantation (Palumbi et al., 2014) and molecular analysis (Courtial et al., 2017) to ascertain the adaptive role.

As regards environmental information, the data we employed had a maximal spatial resolution of ~ 4 km. It is important to acknowledge that crucial drivers of coral survival (heat stress in particular) can vary considerably under the fine‐scale structure (<1 km) of a seascape (e.g. Bay & Palumbi, 2014). Future development of coral seascape genomics should therefore focus on implementing new approaches to describe environmental variation at finer scales (Riginos et al., 2016). For instance, the Landsat 8 satellite (U.S. Geological Survey, 2016) allows to evaluate thermal patterns at less than 100 m of resolution since 2013 (Vanhellemont, 2020) and could therefore represent a valuable input for future studies.

Another element to mention is that we employed a straightforward method to describe coral connectivity in order to facilitate the reproducibility of the analysis. However, there are more sophisticated approaches to describe both genetic and physical distances between reefs that might produce more accurate models of connectivity. For instance, recent works (Matz et al., 2018, 2019) showed that the use of the F_ST_ metric could be replaced with directional estimates of gene flow (Gutenkunst, Hernandez, Williamson, & Bustamante, 2009) and the sea distances could be calculated out of forward‐in‐time dispersal simulations (Lett et al., 2008).

Finally, when calculating connectivity and adaptation indices, we assumed that the demographic and environmental patterns observed at the twelve sampling sites were representative for those of the whole archipelago and that *A. digitifera* were a ubiquitous species. These generalizations might be source of bias in the calculation of the indices. For instance, the twelve sampling sites used in this study cover the higher half of BAF range observed across the Ryukyu Archipelago. Because of this, we might be missing adaptive processes necessary to cope with small to moderate heat stress (i.e. lower half of the BAF range). To avoid this situation in future studies, we suggest to verify these assumptions before starting the seascape genomics study and to define a sampling strategy that minimizes the risks of collecting an unrepresentative data set (Selmoni et al., 2020).

### 5.5 Application in conservation

Conservation policies require objective and quantifiable information to prioritize areas for intervention efforts (OECD, 2017). In this study, we presented an original framework to calculate indices matching these requirements to describe the connectivity and adaptive potential against heat stress of a flagship coral species of the north‐western Pacific. Insights of this kind are essential for effective planning of coral conservation strategies (Baums, 2008; Logan et al., 2014; Palumbi, 2003; Van Oppen et al., 2015).

As they are derived from a universal metric of population connectivity (F_ST_; Weir & Cockerham, 1984), the indices we propose here are computable for any coral species. Thus, connectivity indices for different species can be compared or aggregated for conservation management planning within a region. Furthermore, each of the indices we propose is expressed in a tangible spatial unit (km^2^) that allows for comparison between different datasets and areas.

As an example, the predictions from the connectivity indices can be used to support the planning of marine protected areas (MPAs). An ideal placement of an MPA should ensure that the connectivity to the rest of the reef system is optimal (Krueck et al., 2017; Thomas et al., 2014), and the OCI provides this information (Figure 5b). Furthermore, the computation of the ICI (Figure 5a) from a protected area to the rest of the reef system could be used to compare how different locations of MPAs may modify the connectivity to other specific regions.

Similarly, information on adaptive potential could be used to inform conservation strategies. For instance, an MPA could be established to protect reefs with a high ${\text{PA}}_{\text{heat}}$ (i.e. those likely to carry the traits necessary to persist against heatwaves) from local stressors. Alternatively, this information could support the planning and location of coral nurseries to reinforce the adaptive potential of a population (Baums, 2008; Van Oppen et al., 2015). For instance, this could be done by transplanting corals from reefs with high ${\text{PA}}_{\text{heat}}$ to reef with low ${\text{API}}_{\text{heat}}$ (i.e. reefs that had not experienced heat stress and are less likely to receive heat‐adapted corals via natural migration).

To date, the calculation of these indices can be performed using R scripts and codes (R Core Team, 2009, 2016) made publicly available in this research. In the future, however, this framework should be transposed to a more user‐friendly interface to facilitate its use by conservation managers.

## 6 CONCLUSIONS

This study highlights the value of a seascape genomics approach for supporting the conservation of corals. We applied it to a flagship coral species of the Ryukyu Archipelago and identified genetic variants that may underpin adaptation to heat stress. Coupling this information with a genetic analysis of connectivity made it possible to evaluate the adaptive potential at the scale of the entire study area. The outputs of this analysis are quantitative indices that could be used to support objective prioritization of reefs in conservation plans. This framework is transferable to any coral species on any seascape and therefore constitutes a useful conservation tool to evaluate the genomic adaptive potential of coral reefs worldwide.

## Data Archiving Statement

All the data and codes used in this article are publicly available on Dryad (https://doi.org/10.5061/dryad.qz612jm90).

## Supporting information

Supplementary Material
