# Supplementary material for: Seascape genomics as a new tool to empower coral reef conservation strategies: An example on north‐western Pacific Acropora digitifera
Source: Evol Appl. 2020 Mar 19;13(8):1923–38. doi: 10.1111/eva.12944 (PMC7463334; doi:10.1111/eva.12944)

Supplementary Material

Seascape genomics as a new tool to empower coral reef conservation strategies: an example on north-western Pacific *Acropora digitifera*.

Table of content	
Tab S1	**Environmental Variables included in the Seascape Genomics analysis**
Fig. S1	**F_{ST} analysis by genomic position**
Fig. S2	**Discriminant Analysis of Principal Components (daPCA) of the Genotype Matrix**
Fig. S3	**Significant Genotype-Environment Associations**
Fig. S4	**Connectivity models**
Fig. S5	**Example of dF_{ST} variation across study area**
Fig. S6	**dF_{ST} from Northern Philippines**

Supplementary Table 1. Environmental Variables included in the Seascape Genomics analysis. For each remote sensing product, the table shows the sources (CMEMS= Copernicus Marine Environment Monitoring System; NOAA= National Oceanic and Atmospheric Administration; IEDA= Interdisciplinary Earth Data Alliance) and the corresponding identifier (Product Name). Temporal range and resolution indicate the duration and the frequency of the remote sensing period, respectively. The variables calculated for the seascape genomics analysis are listed in the *Derived Variables* column with the following abbreviations: OM (overall mean), OMsd (overall standard deviation), HM (highest monthly mean), LM (lowest monthly mean), HMsd (standard deviation of the month with highest mean), LMsd (standard deviation of month with lowest mean).

Variable Name	Product Name	Source	Temporal Range	Temporal Resolution	Spatial Resolution	Derived Variables
Sea Surface Temperature	SST_GLO_SST_L4_REP_OBSERVATIONS_010_011	CMEMS	1985-2007	Daily	0.05 °	OM, OMsd, HM, LM, HMsd, LMsd
Sea Surface Salinity	GLOBAL_REANALYSIS_PHY_001_030	CMEMS	1993-2010	Daily	0.083 °	OM, OMsd, HM, LM, HMsd, LMsd
Chlorophyll Concentration	OCEANCOLOUR_GLO_CHL_L4_REP_OBSERVATIONS_009_082	CMEMS	1998-2010	Daily	4 km	OM, OMsd, HM, LM, HMsd, LMsd
Sea Currents Velocity	global-reanalysis-phy-001-030	CMEMS	1993-2010	Daily	0.083 °	OM, OMsd, HM, LM, HMsd, LMsd
Suspended Particulate Matter	oc-glo-opt-multi-l4-spm	CMEMS	1997-2010	Monthly	4 km	OM, HM, LM
Photosynthetically Available Radiations	erdMH1par0mday	NOAA	2003-2010	Monthly	4 km	OM, HM, LM
Bathymetry	Global Multi-Resolution Topography	IEDA	-	-	down to 100 m	Depth
Population Density	SEDAC	Columbia University	2015	-	0.1 °	Population density on buffer 50 km
Bleaching-alert-temperature	Derived from SST		1985-2007	Daily	0.05 °	Frequency
Alkalinity	Derived from SST + pH		1993-2007	Daily	0.05 °	OM, OMsd, HM, LM, HMsd, LMsd

Supplementary Figure 1. Fst analysis by genomic position. The fixation index (Fst) between each pair of sub-populations (a: Okinawa and Kerama, b: Okinawa and Yaeyama, c: Yaeyama and Kerama) was calculated for every SNP in the filtered genetic dataset (graphs on the first column). Fst values were then averaged by genomic position: either by contig (graphs on the second column), non-overlapping 100 kbs window (third column) or non-overlapping 50 kbs window (fourth column).

a) Fst Okinawa – Kerama

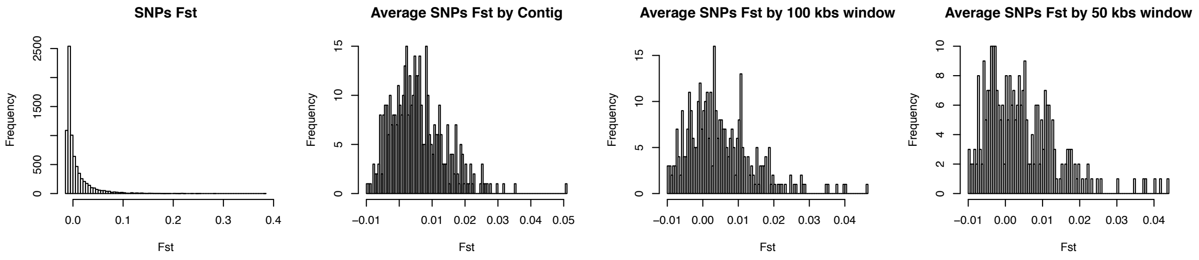

b) Fst Okinawa – Yaeyama

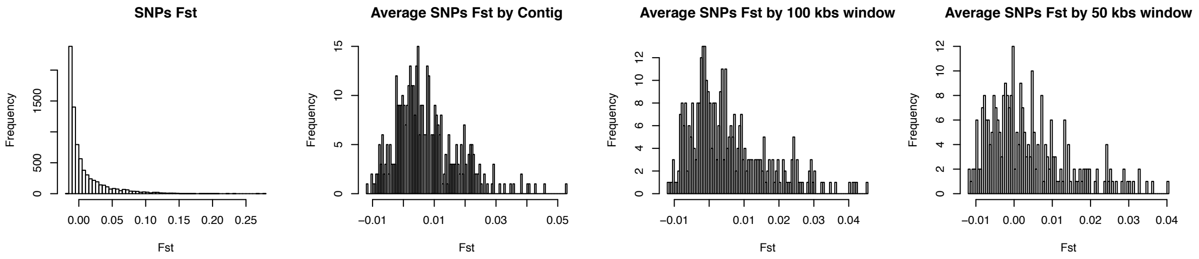

c) Fst Yaeyama – Kerama

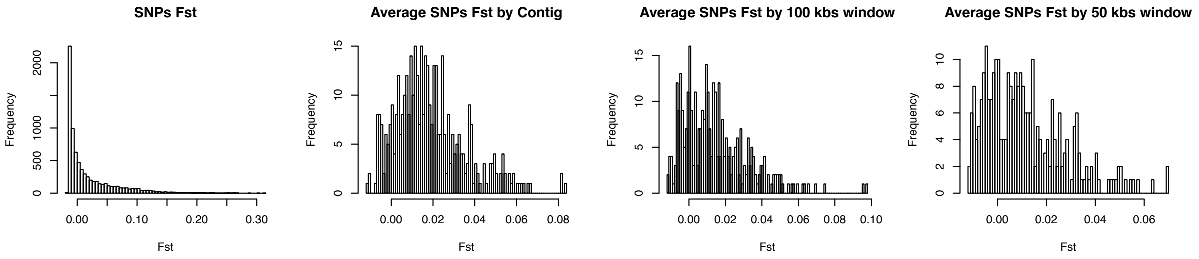

Supplementary Figure 2. Discriminant Analysis of Principal Components (daPCA) of the Genotype Matrix. A daPCA of the genotype table was performed to investigate the neutral structure of genetic variation in the population using the R adegenet package. Graph a) shows the (Bayesian Information Criterion) BIC value against the number of clusters, suggesting the presence of two groups. Graph b) displays that the first discriminant function allows to distinguish these groups and the map in c) shows the average of this value across sampling sites. We can see a strong contrast between sites reefs in the south and those in the North of Okinawa islands, together with those in the south of the Archipelago (Yaeyama).

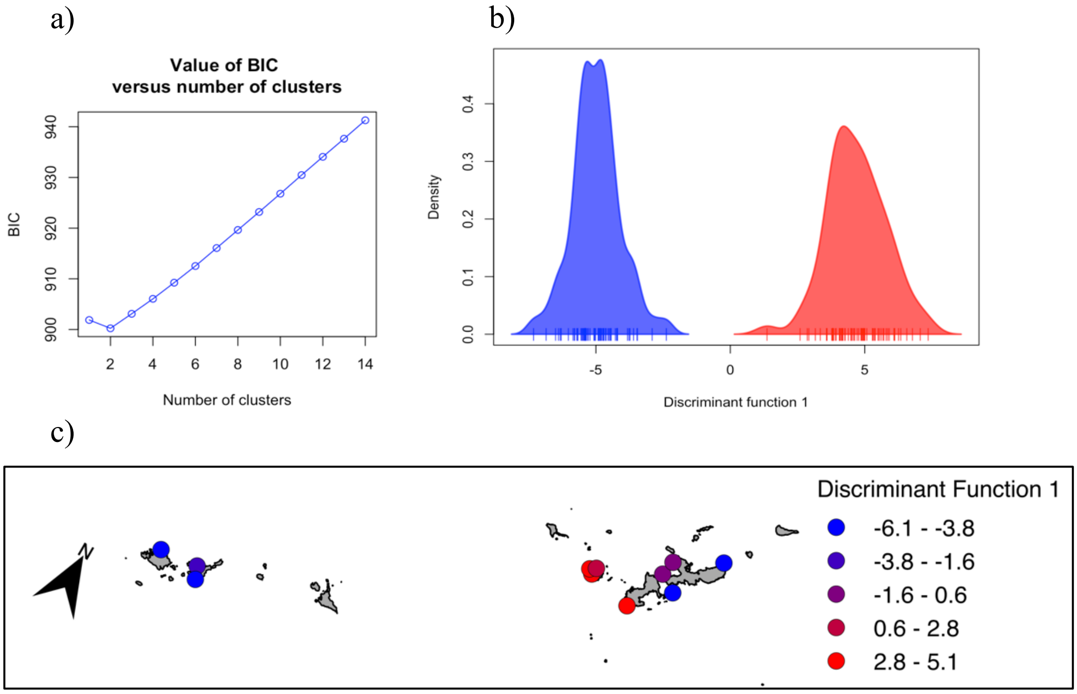

Supplementary Figure 3. Significant Genotype-Environment Associations (SGEA). For every SGEA, three display items are shown: the logistic model linking the genotype frequency with the environmental gradient of interest (left graph); the maps showing the genotype distribution (white points: colony without genotype, black points: colony with genotype) and the environmental gradient (background colors) in the Yaeyama area (central map) and Okinawa (right map). The points on the maps are scattered around the real sampling locations to facilitate the visualization.

SGEA1) NW_015441080.1:208400 vs bleaching alert frequency

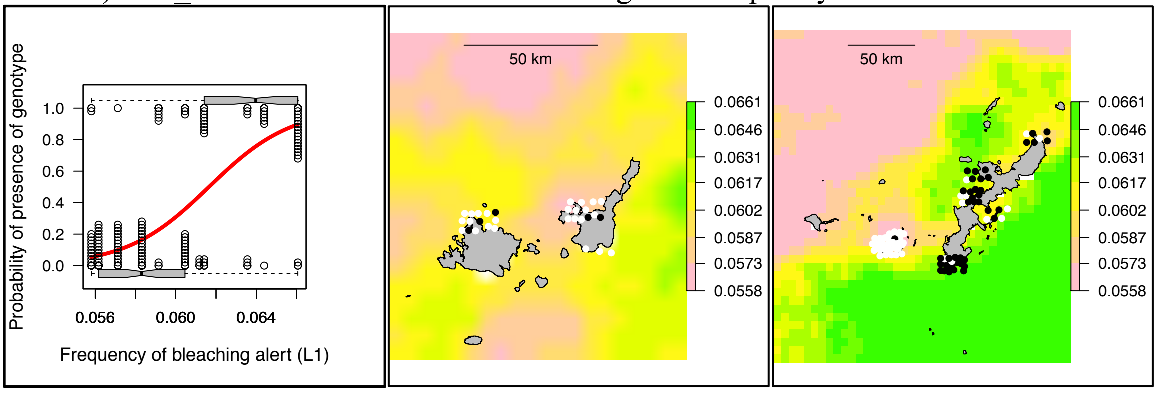

SGEA2) NW_015441080.1:963851 vs bleaching alert frequency

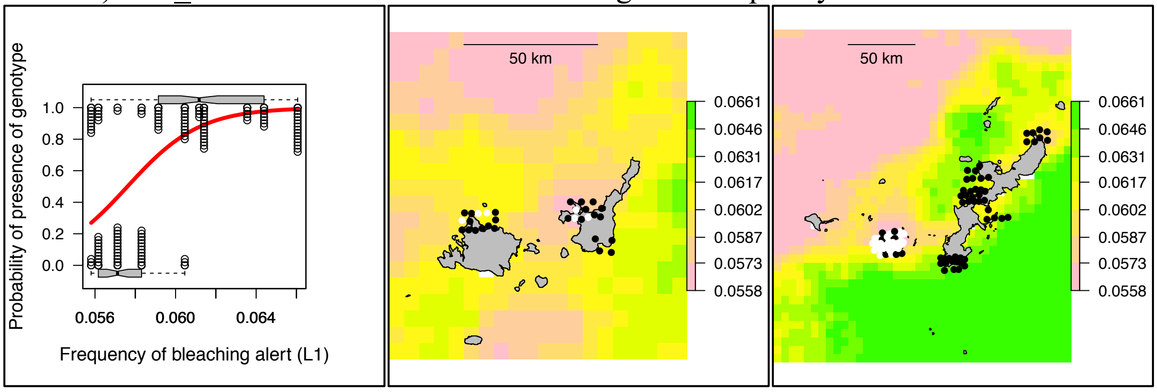

SGEA3) NW_015441121.1:665651 vs bleaching alert frequency

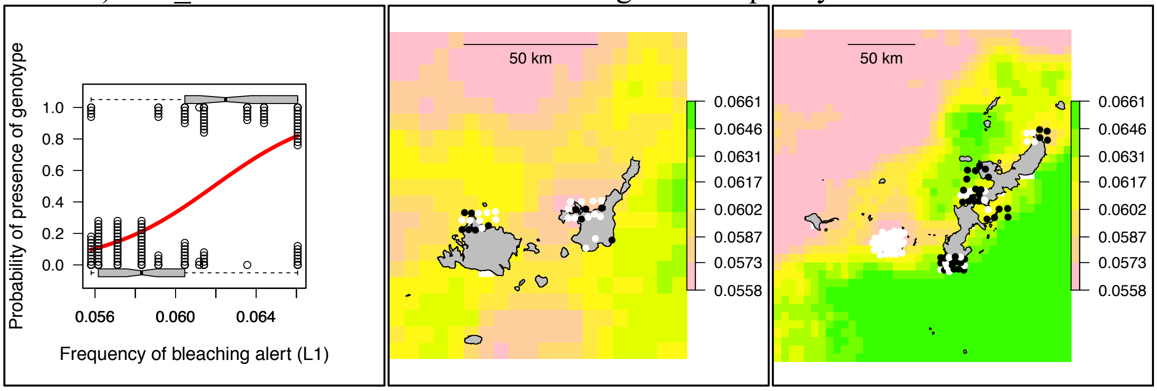

SGEA4) NW_015441261.1:566971 vs lowest average monthly salinity

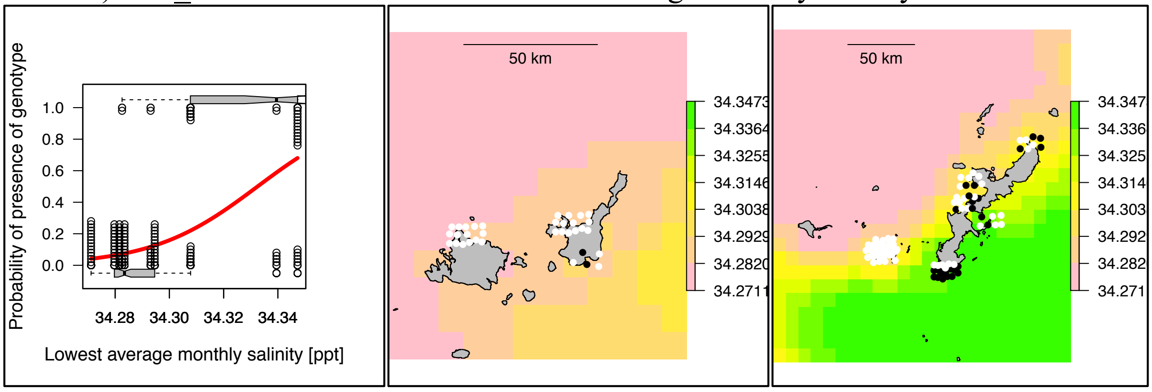

SGEA5) NW_015442197.1:32233 vs bleaching alert frequency

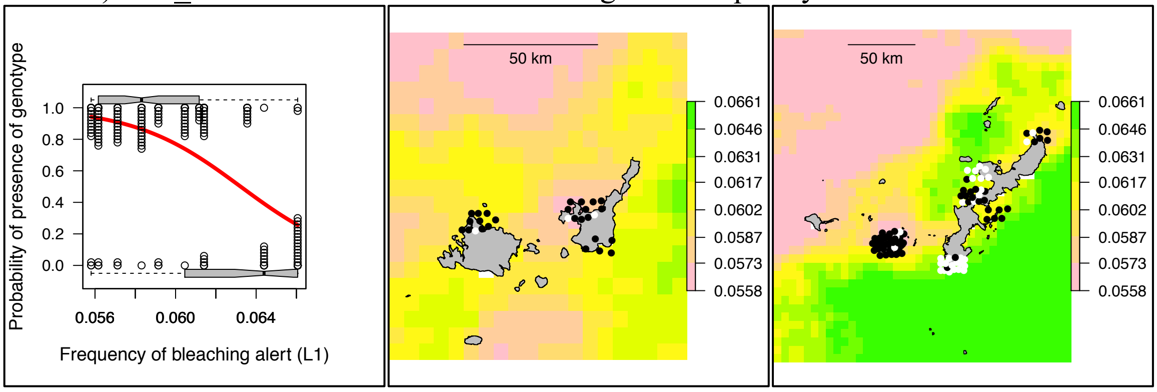

SGEA6) NW_015441195.1:470076 vs bleaching alert frequency

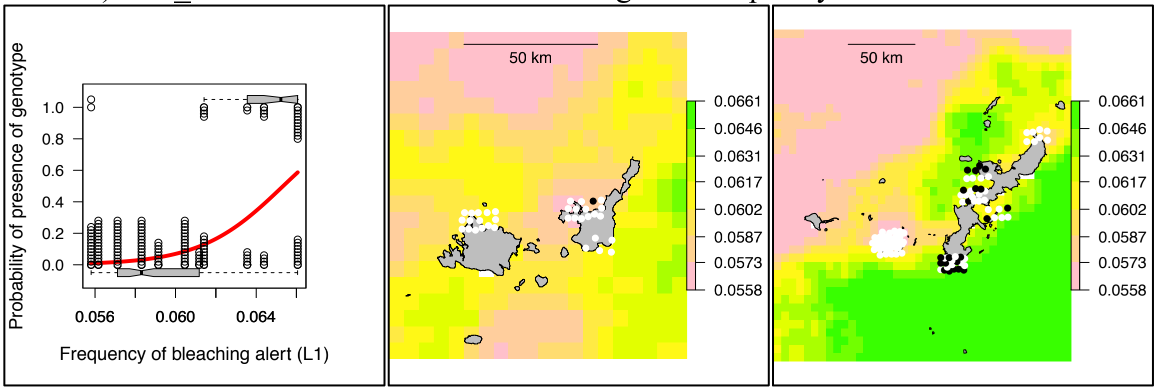

SGEA7) NW_015441282.1:27616 vs bleaching alert frequency

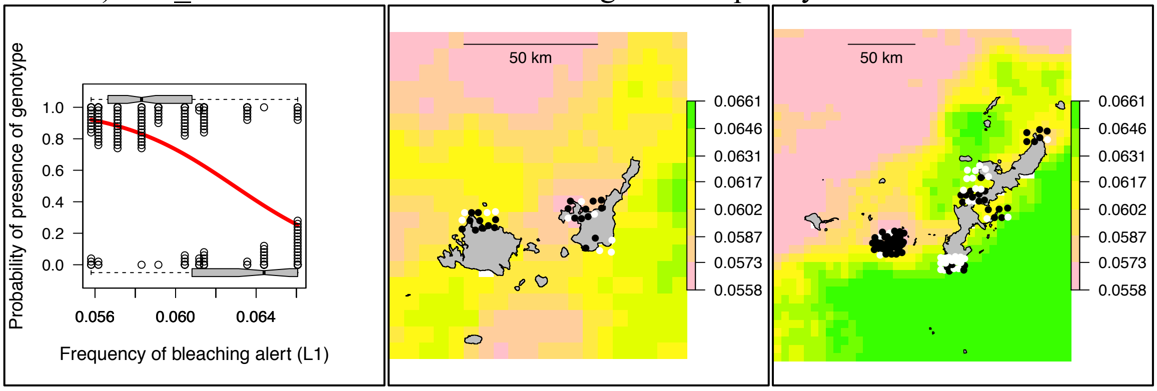

SGEA8) NW_015441785.1:16151 vs bleaching alert frequency

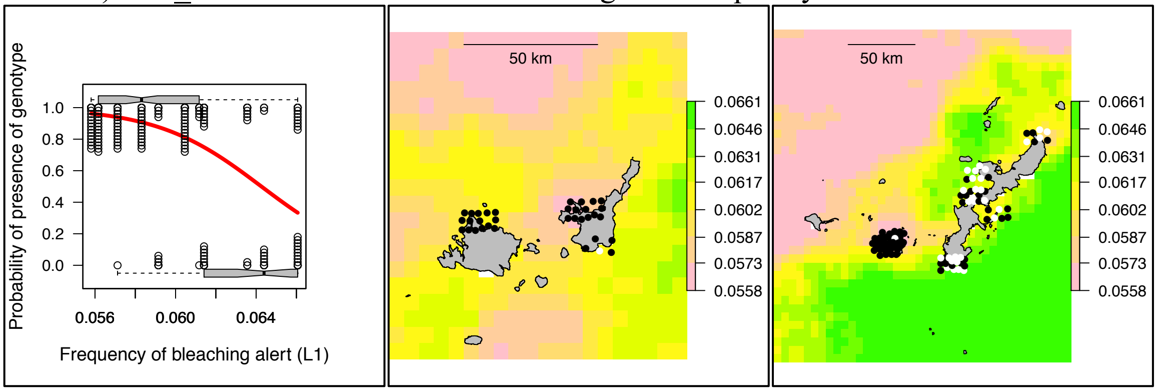

SGEA9) NW_015441192.1:602343 vs lowest average monthly alkalinity

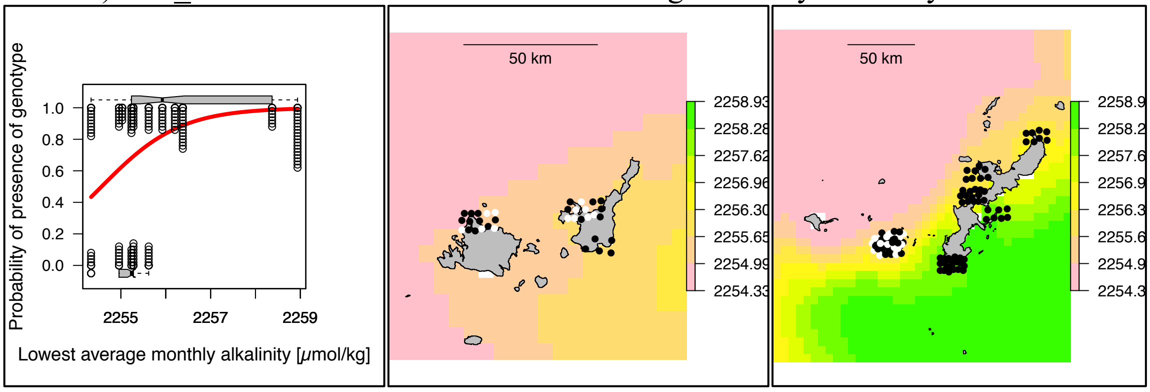

SGEA10) NW_015441113.1:326020 vs bleaching alert frequency

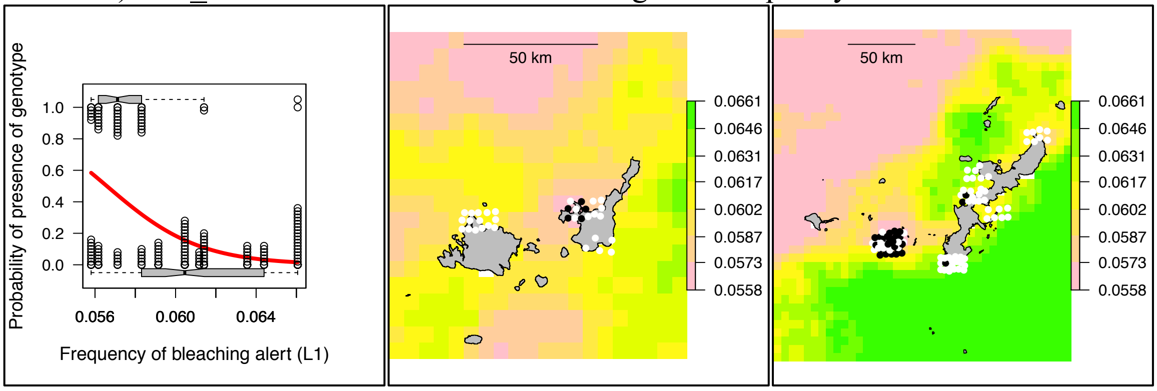

SGEA11) NW_015441391.1:251497 vs bleaching alert frequency

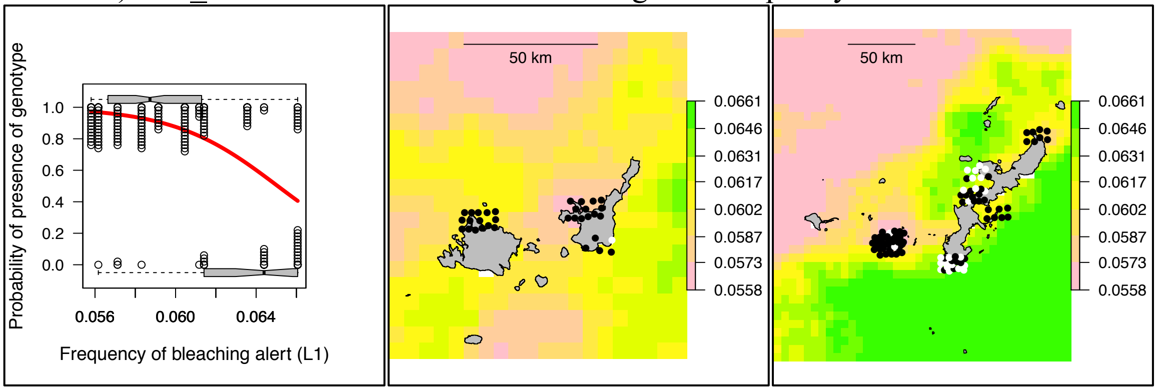

SGEA12) NW_015441600.1:9407 vs lowest average monthly salinity

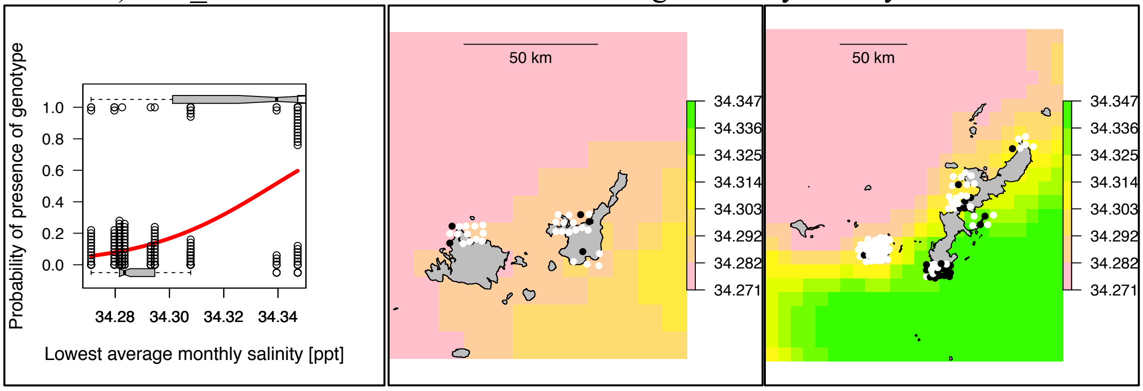

SGEA13) NW_015441190.1:582812 vs bleaching alert frequency

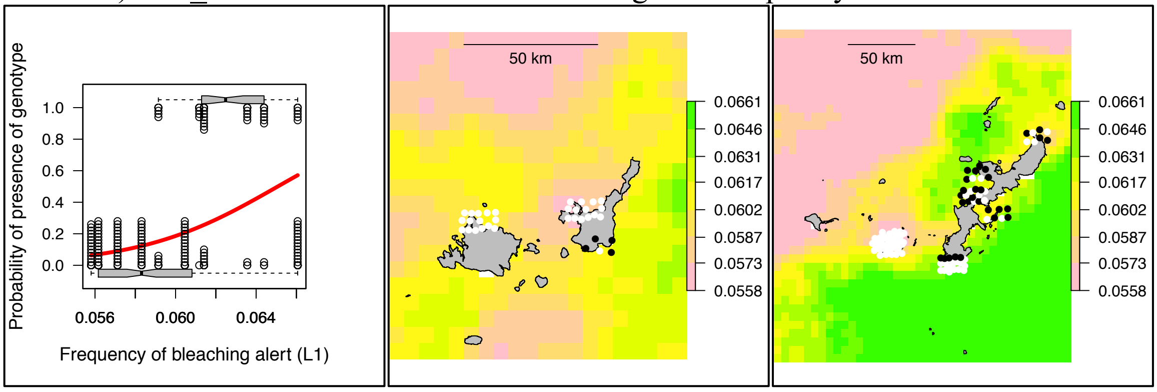

SGEA14) NW_015441072.1:291659 vs bleaching alert frequency

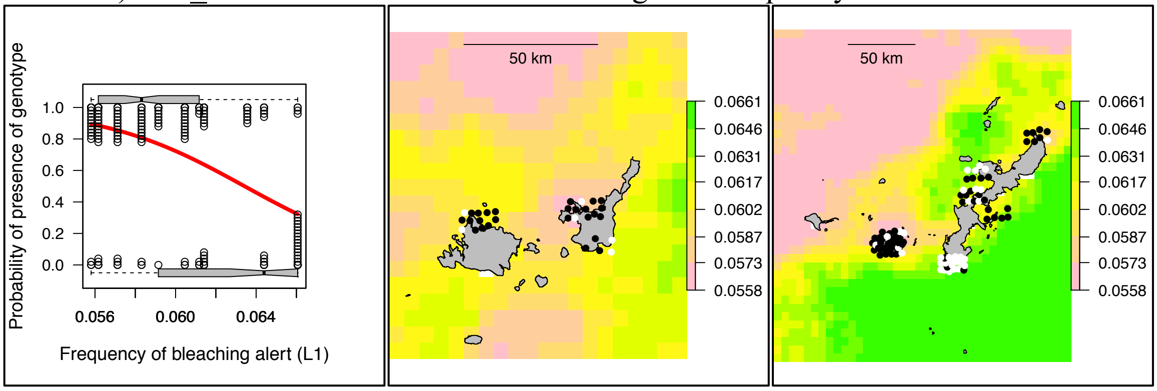

SGEA15) NW_015441328.1:255377 vs bleaching alert frequency

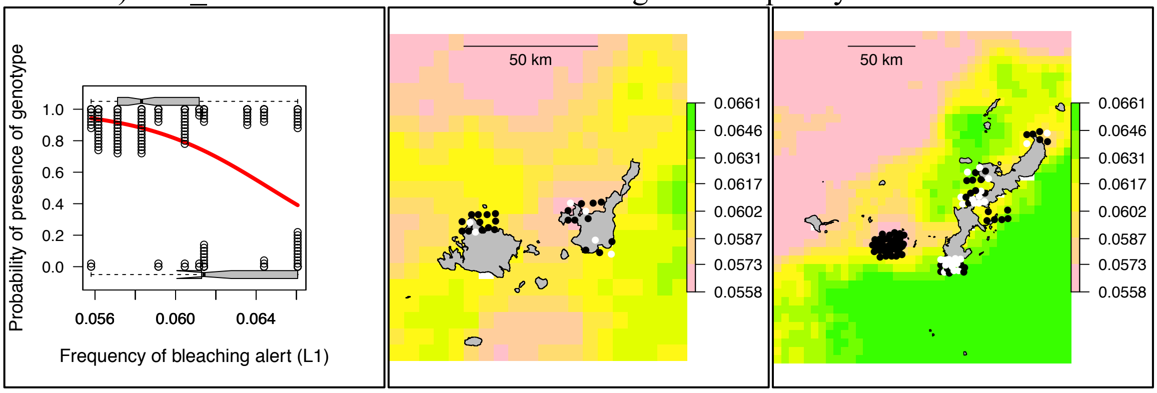

SGEA16) NW_015442007.1:107968 vs lowest average monthly salinity

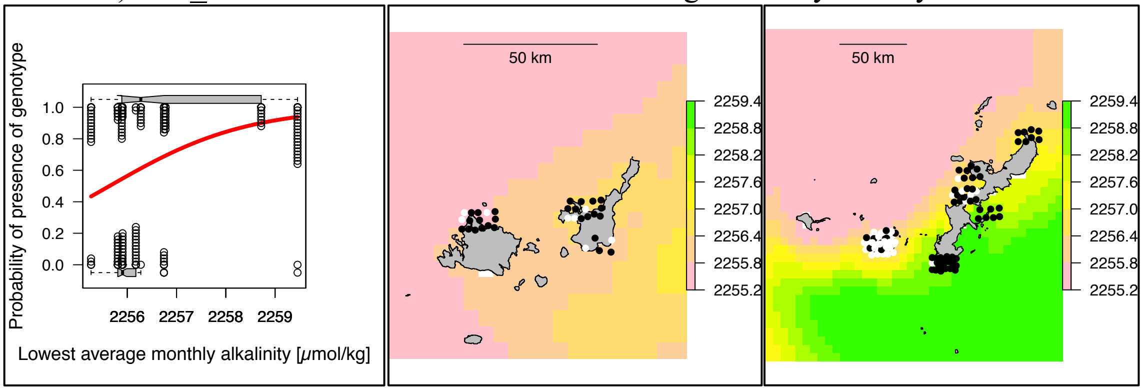

SGEA17) NW_015441133.1:148591 vs bleaching alert frequency

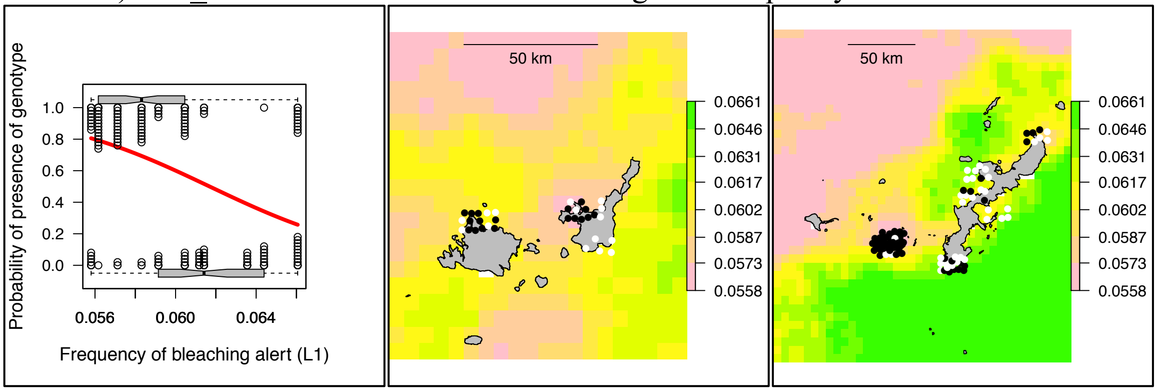

SGEA18) NW_015442144.1:516 vs bleaching alert frequency

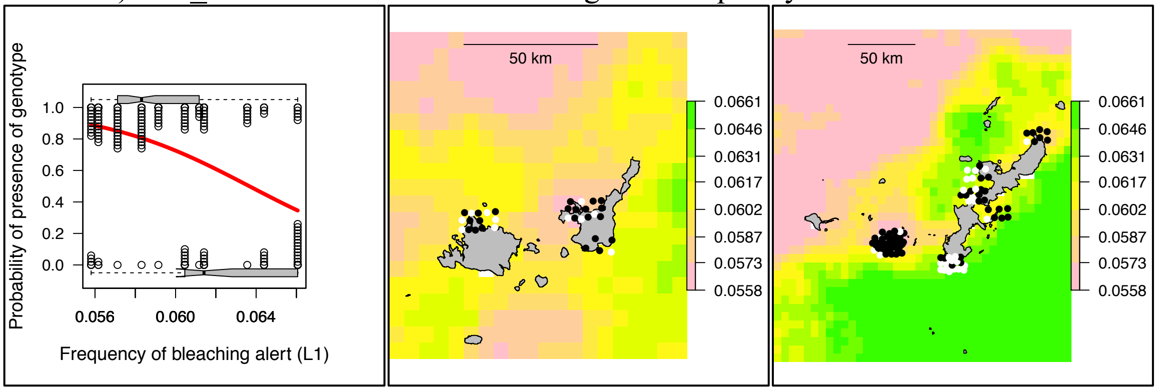

Supplementary Figure 4. Connectivity models. The two models describe the relationship between genetic distance (Fst) and geographic distance calculated in two different ways. In a), geographic distance is sea distance and represents the dispersal costs calculated out of sea current data from the whole study period (1993-2010). In b), geographic distance is the aerial distance between sampling sites.

a)
Fst = -7.253e-04+(SD*1.551e-04)
p = 1.45e-09
Multiple R-squared = 0.7155
AIC = -234

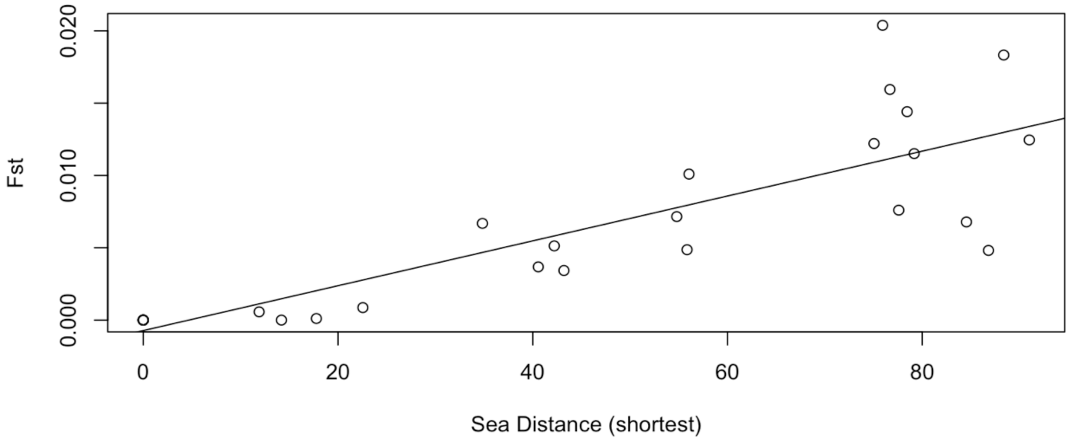

b)
Fst = 0.0016579+(AD*0.0027692)
p = 1.83e-07
Multiple R-squared = 0.66
AIC = -230

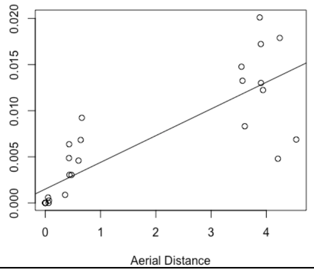

Supplementary Figure 5. Example of dFst variation across study area. The two maps show the dFst values connecting all the reefs of the study area to (a) and from (b) the same reef located in the center of Yaeyama islands (marked with an arrow).

a) dFst to Yaeyama

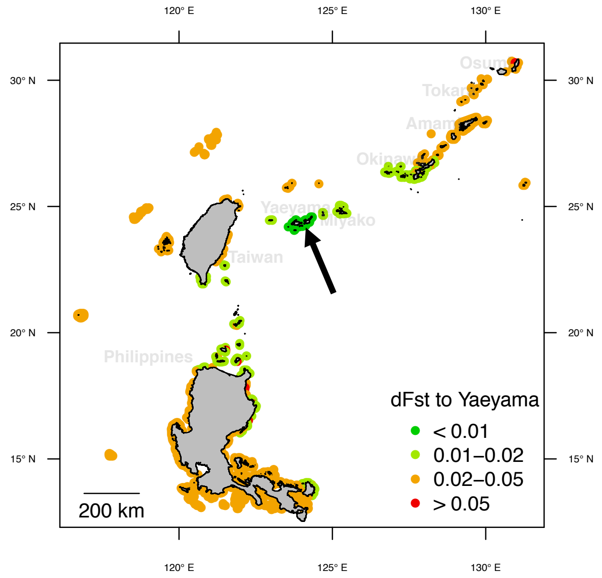

b) dFst from Yaeyama

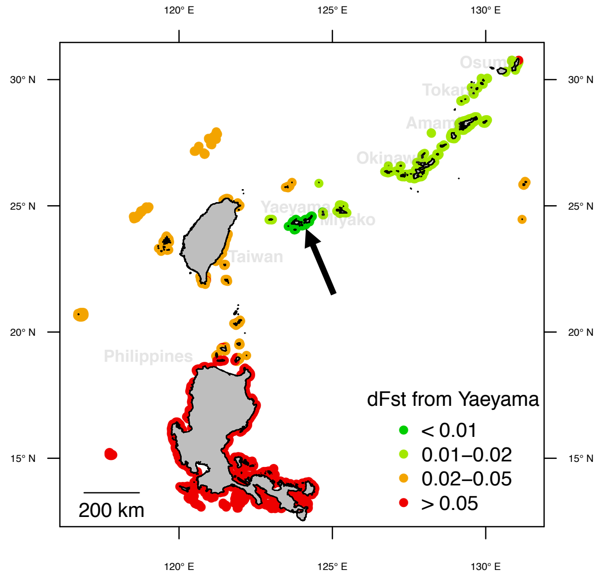

Supplementary Figure 6. dFst from Northern Philippines. The two maps show the dFst values connecting one reef in the north of Philippines (marked with an arrow) to all the reefs of the study area.

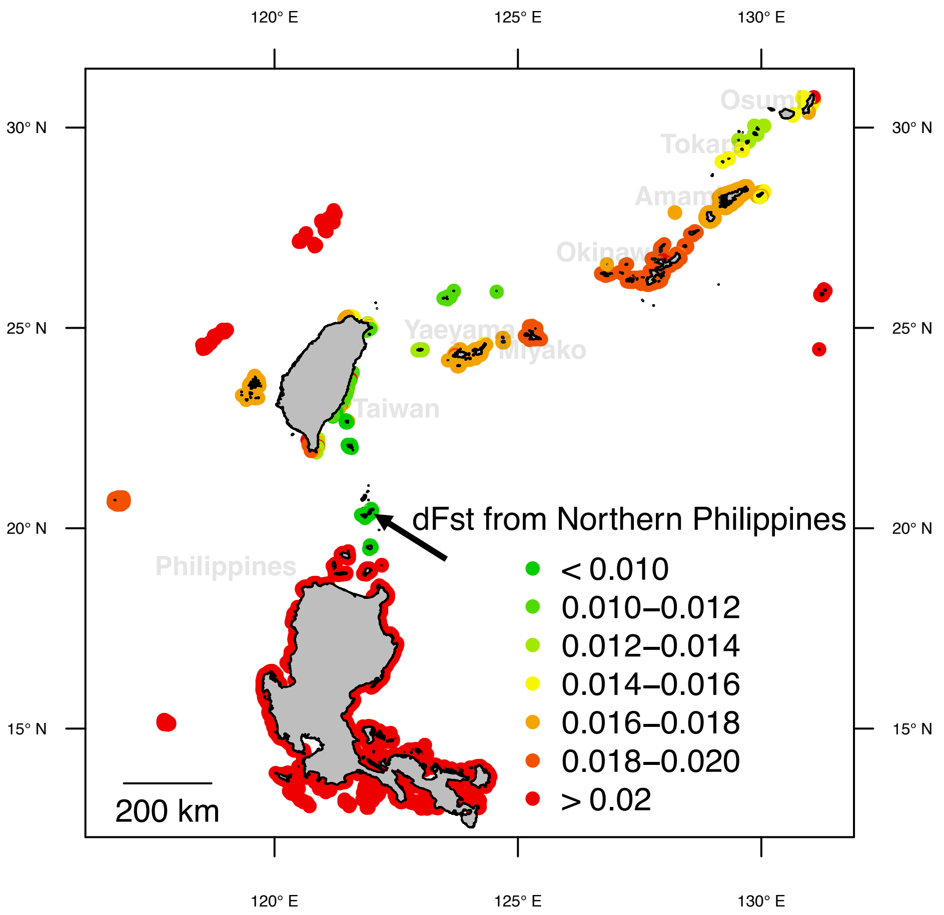

Supplement: Supplementary file 1 — Supplementary Material [file EVA-13-1923-s001.pdf]
